# Comparison of Mediterranean Pteropod Shell Biometrics and Ultrastructure from Historical (1910 and 1921) and Present Day (2012) Samples Provides Baseline for Monitoring Effects of Global Change

**DOI:** 10.1371/journal.pone.0167891

**Published:** 2017-01-26

**Authors:** Ella L. Howes, Robert A. Eagle, Jean-Pierre Gattuso, Jelle Bijma

**Affiliations:** 1 Sorbonne Universités, UPMC Univ Paris 06, CNRS-INSU, Laboratoire d'Océanographie de Villefranche, 181 chemin du Lazaret, Villefranche-sur-mer, France; 2 Alfred-Wegener-Institut Helmholtz-Zentrum für Polar- und Meeresforschung, Bremerhaven, Germany; 3 Université de Brest—UMR 6539 CNRS/UBO/IRD/Ifremer, Laboratoire des sciences de l’environnement marin—IUEM—Rue Dumont D'Urville–Plouzané, France; 4 Department of Atmospheric and Oceanic Sciences, Institute of the Environment and Sustainability, University of California, Los Angeles, CA, United States of America; 5 Institute for Sustainable Development and International Relations, Sciences Po, 27 rue Saint Guillaume, Paris, France; Helmholtz-Zentrum fur Ozeanforschung Kiel, GERMANY

## Abstract

Anthropogenic carbon perturbation has caused decreases in seawater pH and increases in global temperatures since the start of the 20^th^ century. The subsequent lowering of the saturation state of CaCO_3_ may make the secretion of skeletons more problematic for marine calcifiers. As organisms that precipitate thin aragonite shells, thecosome pteropods have been identified as being particularly vulnerable to climate change effects. Coupled with their global distribution, this makes them ideal for use as sentinel organisms. Recent studies have highlighted shell dissolution as a potential indicator of ocean acidification; however, this metric is not applicable for monitoring pH changes in supersaturated basins. In this study, the novel approach of high resolution computed tomography (CT) scanning was used to produce quantitative 3-dimensional renderings pteropod shells to assess the potential of using this method to monitor small changes in shell biometrics that may be driven by climate change drivers. An ontogenetic analysis of the shells of *Cavolinia inflexa* and *Styliola subula* collected from the Mediterranean was used to identify suitable monitoring metrics. Modern samples were then compared to historical samples of the same species, collected during the Mediterranean leg of the Thor (1910) and Dana (1921) cruises to assess whether any empirical differences could be detected. Shell densities were calculated and scanning electron microscopy was used to compare the aragonite crystal morphology. pH for the collection years was hind-cast using temperature and salinity time series with atmospheric CO_2_ concentrations from ice core data. Historical samples of *S*. *subula* were thicker than *S*. *subula* shells of the same size from 2012 and *C*. *inflexa* shells collected in 1910 were significantly denser than those from 2012. These results provide a baseline for future work to develop monitoring techniques for climate change in the oceans using the novel approach of high-resolution CT scanning.

## Introduction

From the start of the Industrial Era to the late 20^th^ century, combustion of fossil fuels and cement production has released approximately 395 ± 20 Pg carbon into the atmosphere, this has been exacerbated by change in land usage which has added a further 185 ± 65 Pg [1]. During 2014, atmospheric CO_2_ levels reached 400 ppmv (http://www.esrl.noaa.gov/gmd/ccgg/trends/), compared to a pre-industrial level of 278 ppmv [1]. The oceans have absorbed approximately 28% of total anthropogenic CO_2_, causing changes to the carbonate chemistry by decreasing pH, carbonate ion concentration, and thus, the saturation state (Ω) of calcite and aragonite [2, 3]. Lowered saturation state and pH can make the secretion of calcium carbonate skeletons more problematic and can increase dissolution of calcium carbonate structures [4].

As it is a semi enclosed system with a short water mass residency time, the Mediterranean Sea is reactive to external forcing and has been highlighted as a “hotspot” for climate change [5]. Long term studies observe a shift to a warmer drier system [6] with evidence from time series and satellite data showing a steady increase in SSTs since the year 1900 at a rate of 0.03–0.167°C yr^-1^ [7]. Under RCP8.5, SSTs are projected to increase by 4.08°C between 2010 and 2099 [8]. Despite having higher alkalinity, the Mediterranean is acidifying at the same rate as the open oceans, deep waters decrease in pH by -0.005 to -0.06 pH units yr^-1^, with the highest rates of acidification in the western basin [9, 10]. The saturation states of calcium carbonate are above 5 and 3, respectively for calcite and aragonite [11] They could decline by about 30% by the end of the century [12].

Monitoring the extent of medium to long term climate and anthropogenic changes in a specific area, such as the Mediterranean, can be challenging and requires the use of time series that are scarce and costly to maintain. Another option is to use sentinel or indicator species, which are known to be sensitive to certain environmental changes and can be examined for early deleterious effects of acidification before they are likely to become apparent in other species.

Thecosome pteropods are a group of holoplanktonic opistobranchs, found in all the world’s oceans [13]. The Mediterranean Sea hosts a diverse range of tropical and temperate species [14], including the two tropical species that will be the focus of this study, *Cavolinia inflexa* (Cavoliniidae) and *Styliola subula* (Cresidae). Thecosome pteropods produce thin aragonitic shells that serve as protection from predators and parasites as well as providing ballast and stability in the water column [13]. The shells of pteropods have adapted to suit their pelagic lifestyle, they are very small; < 1 mm to 15 mm in diameter and are extremely thin, ranging from about 6 μm to 100 μm in thickness [13]. As the only pelagic aragonite precipitators, pteropods are important contributors to the biogeochemical cycling of carbon [13]. In the northwestern Mediterranean, during periods of high carbonate flux, pteropods shells are a major constituent and have been identified as an important part of the total mass flux in the area [15, 16]. Pteropods also act as an important link in ocean food webs, exhibiting a top down control on smaller zooplankton and phytoplankton as well as being important prey for a wide variety of organisms including larger zooplankton, fish, marine birds and mammals [13, 17]. In the north western Mediterranean, during seasonal pulses of high abundance, *C*. *inflexa* can occur in numbers as great as 900 ind m^-3^ [18], in the western basin, *S*. *subula* are less abundant than *C*. *inflexa*, peaks of up to 240 ind m^2^ have been recorded [19]; however, despite their abundance, their role in the Mediterranean trophic web is not well defined. Both species can be found over varying depths, *C*. *inflexa* are classed as epipelagic and *S*. *subula* as mesopelagic (maximum 200–500 m) [14] and the two species have been shown to display similar vertical distributions [19]; both undertake diurnal vertical migration [19, 20].

Because of their extremely thin shells, and due to aragonite being more soluble than calcite [21], pteropods are thought to be particularly sensitive to the effects of climate change; at risk, not only from warming and associated phenomena, but also ocean acidification. Experimental work has shown pteropods to be sensitive to projected future conditions, exhibiting decreased calcification rates and increased shell dissolution with declining pH and increasing temperatures [22–25]. They are currently one of the only organisms to exhibit signs of acidification effects in natural populations [26–28] and their long term abundance has been demonstrated to be sensitive to temperature variations [29]. Experimental work with pteropods has mostly focused on high latitude species; however, a few studies have investigated impacts on temperate and tropical species, including those found in the Mediterranean. Long-term population abundances in the Mediterranean have been shown to fluctuate with temperature, not yet displaying any signs of a deleterious effect of ocean acidification on abundance [18]; although, experimental work has shown larval stages of *C*. *inflexa* to be sensitive to acidification effects, developing without a shell when raised in undersaturated conditions [23]. A recent study undertaken in Australian waters used two tropical pteropod species, also found in the Mediterranean, to assess long-term effects of ocean acidification on pteropods by comparing a collection of historical samples to modern specimens of the same species [30]. The authors found changes in shell structure over the latter half of the 20^th^ century in *Creseis clava* (referred to therein as *Creseis acicula*) and *Diacavolinia longirostris*, with an observed decrease in shell thickness in parallel with declining environmental pH. Due to their wide distribution and apparent sensitivity to future climate conditions, pteropods have been suggested as a potential indicator species, with OSPAR/ICES recommending the extent of pteropod shell erosion as a proxy for monitoring ocean acidification [27, 31]. Shell erosion is an excellent metric for acidification in high latitude and upwelling areas that already experience periods of undersaturation; however, it cannot be applied to supersaturated basins such as the Mediterranean.

Despite the maintenance of supersaturated conditions in the Mediterranean, the decline in pH and increase in temperature could still prove problematic for sensitive species such as pteropods, thus, it is necessary to gather more information on any effects of climate change stressors on Mediterranean pteropods and find new methods to monitor changes in these sentinel species. The work of Roger et al. [30] demonstrated that, even in supersaturated conditions, there might be changes to pteropod shell thickness. Here, we test the potential for using shell thickness as a indicator of climate change stressors by comparing modern and historical samples of two Mediterranean species (*Cavolinia inflexa* and *Styliola subula*) and searching for empirical differences in shell thickness, density and microstructure, to provide a baseline for future monitoring work. In order facilitate an accurate comparison of the historical and modern samples; an investigation of the relationship between shell biometrics and ontogenetic stage in modern pteropod samples was undertaken in parallel. We will use the novel method of X-ray computed tomography (CT) imaging. This method has the advantage of being non-destructive and gathering a large quantity of data that would not be available using other methods [32]. In recent years, the technique has begun to be applied to studying the effects of ocean acidification on calcifying organisms such as foraminifera [33], corals [34], fish (otoliths) [35] and coralline algae [36]; however, to the best of our knowledge, this is the first time this approach has been used on pteropods. Additionally, previous studies have utilized lower resolution “micro-CT scanners” [33] than the instrument capable of sub-micron imaging utilized here. This study will also provide a “snapshot” to the long term implications of anthropogenic CO_2_ on these two Mediterranean pteropod species.

## Materials and Methods

### Sample collection

**Fig 1:**
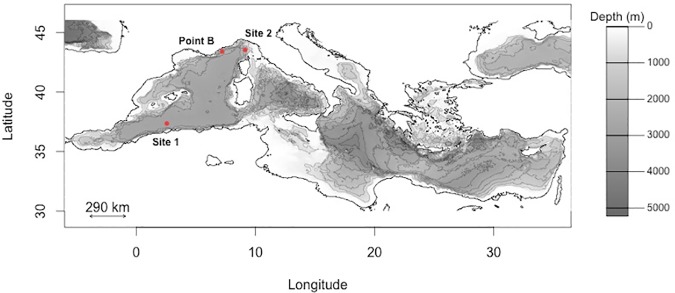
Sampling locations for the historical samples of *Cavolinia inflexa* and *Styliola subula* and for the modern collections of both species (Point B).

No field permit was required for this work and the fieldwork did not involve endangered or protected species. All historical samples were preserved in 90% ethanol and donated from the private collection of Dr Jeannine Rampal. Historical samples of *C*. *inflexa* were sampled by vertical net tow at station 125 (43.54° N, 7.19°E) of the Thor Expedition in the year 1910 [37]. Historical samples of *S*. *subula* were sampled by vertical net tow at station 1124 (37.15°N, 2.55°E) of the 1921, Dana expedition [38]. Modern specimens of *C*. *inflexa* and *S*. *subula* were collected from Point B, Villefranche-sur-Mer, 43.41°N, 7.19°E (Fig 1) between August 2011 and August 2012. Sampling was undertaken with a 1 m mouth diameter, “Regent” plankton net (mesh size 680 μm), towed obliquely from 70 m depth, very slowly to avoid shell damage. Samples were immediately fixed and stored in in 90% ethanol.

### CT scanning

A total of 89 *C*. *inflexa* (30 historical samples and 59 modern samples) and 84 *S*. *subula* (27 historical samples and 57 modern samples) specimens were selected for CT scanning. X-ray computed tomograpahy (CT) imaging was carried out with a General Electric Company Phoenix Nanotom S Instrument. CT scanning with this class of instruments is sometimes termed “nano-CT”, as they are theoretically capable of imaging in the submicron range, however, typically operate at a resolution of 1–10 μm. Samples were analysed in pairs using a custom fashioned balsa wood mount, alongside a calcite rhomb that acted as a reference material and was present in every scan. Two pteropod samples (one historical, one modern) and the calcite standard were typically loaded in each mount. Additionally a “standard pteropod” of the species *S*. *subula* was analysed multiple different times to assess the reproducibility of our analyses. Sample mount, X-ray source and detector geometry were positioned to maximize resolution and were kept constant throughout all analysis of samples of a particular species. An X-ray tube voltage of 80 kV and current of 100 mA were used, without any physical x-ray filtering with metal strips. The detector is a proprietary high-contrast CT scanner detector from GE technologies, 12 bit, with 3 x virtual detector enlargement (max. 6,900 pixel detector width). Full instrument specifications can be found at https://www.gemeasurement.com/sites/gemc.dev/files/nanotom_brochure_english_0.pdf. A voxel (a 3D unit of space which varies in dimensions between CT reconstructions depending on scanning parameters) size of <5 μm^3^ was typically achieved using this setup. Scan setup and assembly of 3D images was carried out with proprietary Datos/x 2.0 software. One thousand individual X ray absorption profiles of each mount were taken from marginally different orientations as the sample was rotated on the mounting stage, and combined to build a 3D rendering of the image using Datos/x 2.0.

**Fig 2:**
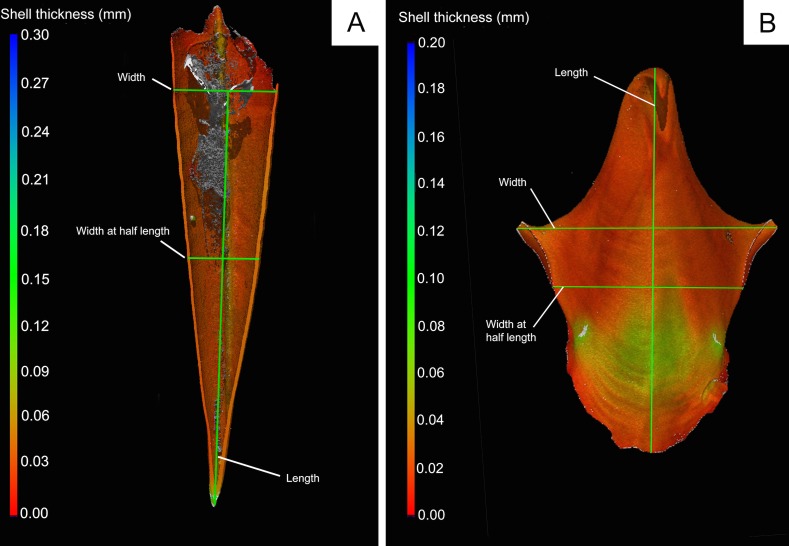
Three Dimensional CT renderings of A. *Styliola subula* (in cross section) and B. *Cavolinia inflexa*. Colouring indicates assessment of shells thickness at a particular point via standard “wall-thickness” analysis with VGStudio MAX, with thickness scales given to the left of each panel. Also indicated, as green lines are dimension measurements taken from the CT renderings. The grey area inside the *S*. *subula* is organic material and the lack of colouring indicates that it has been excluded from the thickness determination.

Image analysis was carried out with Volume Graphics VGStudio MAX version 2.2 software. Initial 3D renderings contained two pteropods and a calcite standard, which comprised the objects in each balsa wood mount. The 3D image was then split into separate renderings for each object for analysis as illustrated in the S1–S4 Figs. We found that a manual surface determination enabled the hard calcium carbonate shell to be separated from organic material comprising the soft-body of the pteropods and also separated the pteropod shell from the balsa wood mount without clipping the pteropod shell rendering. The differential X-ray absorption of organic matter, balsa wood, and calcium carbonate translates into different greyscale intensities in the reconstructed 3D image; therefore, it was possible to filter out balsa wood and organic material from the analysed data for volume, wall thickness etc. This process is illustrated with text in S1–S4 Figs. Object volume, average thickness and surface area were determined using standard Volume Graphics analysis. In addition, dimension measurements were taken on 3D renderings of shells. For *C*. *inflexa*, three dimension measurements were taken, the width (at widest point), the width at half shell length and length (Fig 2A). For *S*. *subula*, the dimension measurements taken were the total length, width (diameter at widest point) and the “diameter at half length” (Fig 2B). Reproducibility was assessed by replica analysis of the carbonate rhomb standard and an individual *S*. *subula* specimen that was analysed multiple times. CT based measurements were compared to measurements taken with a light microscope, which gives an opportunity to assess potential biases in both approaches (S5 Fig). Individual standard analyses are given in S2 and S3 Tables. Average values for the pteropod standard +/- one standard deviation are from n = 15 analyses. Average thickness (μm): 40.8 +/- 3.6, volume (mm^3^): 0.943 +/- 0.176: Surface area (mm^2^): 53.702 +/- 10.1. The data indicate that for these samples the surface area determination is the least precise and the volume the most precise. Calcite rhomb data were used to assess the precision of dimension measurements, in particular. Average standard data from n = 28 analyses. Average Volume = 4.12 +/- 0.07. Dimension 1: 2.00 +/- 0.05, Dimension 2: 1.99 +/- 0.05.

### Shell weight

To reduce the risk of compromising the structural integrity of the shell, organics were not removed until after scanning was complete. Organic material was removed from the shell by soaking in 3:2 ratio of sodium hypochlorite (13%) and deionised water. Once the organics were completely dissolved, the empty shells were rinsed with deionised water and placed in the oven at 50°C for 8 hours, followed by 24 hours in a dessicator. Each shell was individually weighed using a Mettler Toledo microbalance (precision = 0.1 μg). Shell density measurements were determined by calculating calcium carbonate volume from the CT scan derived parameters of surface area and average thickness (Eq 1, where ccV = calcium carbonate volume, sa = surface area, t = thickness). Density was derived from the calculated calcium carbonate volume and the manually measured shell mass (Eq 2, where ρ = density, ccV = calcium carbonate volume, m = mass).

ccV=saxt (1)

$$\rho =\frac{m}{ccV}$$ (2)

### Scanning electron microscopy

Subsamples of 10 adults each from the historical and modern specimens of both species were inspected for signs of dissolution on the outer shell surface. As recommended by Bednaršek et al. [27], the specimens had been previously bleached (described above) to remove the outer organic layer. A further subsample of 10 adult specimens from each sample group were embedded in resin (Araldite 2020) then successively ground with P1200/P2400/P4000 grinding paper (Buehler) and water to dorsal—ventral cross sections along the midline. The samples were then polished, first with 3 μm Buehler diamond polycrystalline suspension then 0.3 μm aluminium oxide suspension. The embedded samples and those prepared for inspection of the outer shell surface were mounted on 1.8 cm (diameter) scanning electron microscopy (SEM) stubs. Specimens were coated with gold palladium for imaging using a Fei Quanta 220 F SEM with operating parameters of 10 kV, spot size 3 and 10 mm working distance.

### Sample selection

In order to differentiate between changes in shell biometrics occurring as a result of environmental influence and those that are part of normal growth, an ontogenetic study was performed. This was undertaken by analysing a wide size range of modern shells to assess changes in shell biometrics that occur at different ontogenetic stages. Modern *C*. *inflexa* samples ranged in length from 2.3 to 6 mm and modern *S*. *subula* samples ranged in length from 2 to 7.7 mm (see S1 Table). For the comparison of the historical to modern samples, the maximum and minimum widths and lengths of each species of the historical sample collection was found. For historical *S*. *subula*, lengths ranged from 4.1 to 6.6 mm and widths from 1 to 1.6 mm, historical samples of *C*. *inflexa* ranged in length from 5 to 6.2 mm and in width from 4.8 to 3.3 mm. Modern samples were then filtered, by species, to exclude samples outside of this size range (see S2 Table).

### pH hindcast

The environmental times series run at Point B from Villefranche-sur-Mer *Service d’Observation en Milieu Littoral* (SOMLIT/CNRS-INSU) was used for pH estimation using the methods detailed in Howes, Stemmann (18). Weekly temperature and salinity measurements have been undertaken since 1957. Since 2007, pH_T_, dissolved inorganic carbon (*C*_T_) and total alkalinity (*A*_T_) have also been measured (data provided in the S1 Table). Water for the determination of dissolved inorganic carbon (*C*_T_) and total alkalinity (*A*_T_) was collected from 10-L Niskin bottles, transferred to combusted glass bottles, overfilled, and poisoned with HgCl_2_ as recommended by Dickson et al. (2007). *C*_T_ and *A*_T_ are determined potentiometrically [39, 40]. Further information on environmental sampling methods is provided at the SOMLIT website (http://somlit.epoc.u-bordeaux1.fr/fr/).

From the SOMLIT time series, we were able to constrain a model for pH hindcasting. Total alkalinity (*A*_T_) was calculated from monthly averages of temperature and salinity using a variation on the equations provided by Lee et al. [41] (Eq 3), where: *A*_T =_ total alkalinity (μmol kg^-1^); S = salinity, T = temperature and a, b, c, d, e are constants.

$$A_{T}=a+b\times S+c\times S^{2}+d\times T+e\times T^{2}$$ (3)

The most parsimonious model was determined from Eq 3 using the Akaike information criterion measure [42] on subsampled (one per 10 weeks, to reduce the autocorrelation of the time series) alkalinity measurements ranging from 2007 to 2012. The temperature variables were dropped as they did not affect the goodness of fit of the model to the measured data. The constants a, b and c were then determined with all measurements from 2007–2012. The coeffcients a, b and c were derived from the weekly measurements of total alkalinity taken between 2007 and 2012. Finally, the model was applied to annual averages of temperature and salinity from 1899–2012.

The SOMLIT time series only extends back as far as the 1950’s, which was insufficient to ascertain an estimate of pH conditions at the time of collection of the historical samples (1910 and 1921). SOMLIT data was used for hindcasting as far as 1957; for data prior to the start of the SOMLIT series, annual temperature values corresponding to the coordinates of sites 1 and 2 were taken from gridded sea surface temperatures (SST) from the Hadley Data Centre [43] (http://rda.ucar.edu/datasets/ds277.3.). Salinity data were unavailable for the full period so values were extrapolated from SOMLIT data as the change in surface salinity in the western Mediterranean has been shown to be roughly linear since the year 1900 [7]. Measured and calculated values of alkalinity for the period of 2007–2012 were compared to assess hindcast accuracy.

The partial pressure of CO_2_ (pCO_2 sw_) was estimated from temperature and concentration of CO_2_ in the atmosphere (CO_2 atm_, ppm; Eq 4.) [44].

$${{pCO}_{2}}_{sw}=a\times T+{{b\times pCO}_{2}}_{atm}+c$$ (4)

There is no measurement of CO_2 atm_ in the Mediterranean region covering the entirety of the study period. The Mauna Loa (19°53’N– 155°57’W, Hawaii) atmospheric CO_2_ time series was used, as this is the only station in the northern hemisphere that has data for the entire study period. To account for geographical differences, the data were compared to the 1979–1997 CO_2 atm_ time series from the Italian station, Monte Cimone (44°18’N– 10°7’E). Seasonal differences were minimised by lagging the Manuna Loa data by 2 months and adjusting seasonal maxima and minima of the values from the Mauna Loa time series to obtain the best fit with the data from Monte Cimone. The coefficients were determined using a linear model for 1 m depth based on pCO_2 sw_ computed from SOMLIT data from weekly measurements of dissolved inorganic carbon and total alkalinity ranging from January 2007 to March 2012. For values before the start of the Mauna Loa time series (pre 1958) values was taken from the Siple ice core dataset [45]. Values of pCO_2 sw_ for the period 2007–2012, derived using hindcast methods were compared with those from the same period that were calculated using the R package seacarb [46] from measurements of *C*_T_ and *A*_T_.

Using the estimations of *A*_T_ and pCO_2 sw_ alongside the measured values of temperature, salinity and pressure, it was possible to calculate seawater pH_T_ using the R package seacarb [46]. Weekly measurements of pH at Point B began in 2007. These measurements (2007–2012) are used to assess the goodness of fit for the calculated data.

### Statistical analysis

All statistical analyses were carried out using R [47]. For direct comparisons between shell thickness, weight and density of the old and new samples, a size filter was applied to the dataset and only individuals of the same length were used. The data were non-parametrically distributed so the Mann Whitney U test was used to analyse differences between the two groups. For the examination of the ontogenetic shell biometrics, a larger set of modern samples was used, which included a range of ontogenetic stages. For this aspect of the analysis, no length filter was applied.

## Results

**Fig 3:**
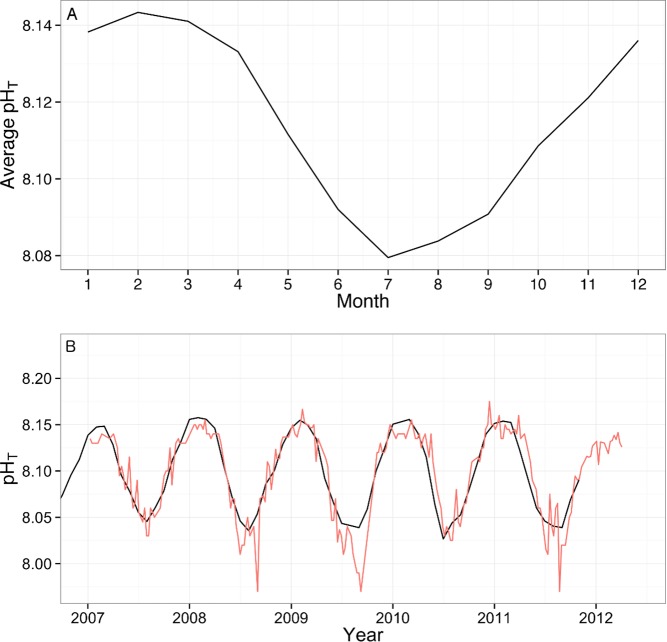
Fig 3 (A) Average annual variation pH_T_ of measured data from SOMLIT time series at Point B from 2007–2012. (B) Comparison of measured pH_T_ values at Point B, Villefranche-sur-Mer (red), to pH_T_ calculated from temperature and salinity values taken from the SOMLIT times series at Point B and the pCO_2 atm_ time series at Mauna Loa (black).

**Fig 4:**
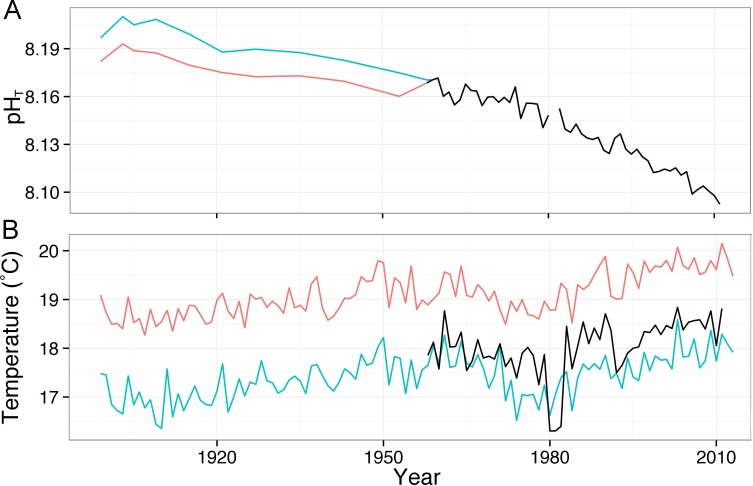
Changes in average pH_T_ (A) and temperature (B) at the collection sites. Red lines show the changes in pH and temperature at site 1, blue lines show changes in pH and temperature at site 2, black lines show changes in temperature and pH at Point B. Changes in pH are calculated by hindcasting using temperature (Hadley SST and Point B time series), salinity (Point B time series) and atmospheric CO_2_ measurements (Mauna Loa time series and Siple ice core data).

The measured pH_T_ data for years 2007–2012 display a seasonal oscillation with highest values in winter months (Feb/Mar) and lowest values in summer (Jul/Aug), the average seasonal variation over the 5 year time series is 0.07 ± 0.05 pH units (Fig 3A). Comparison of calculated pH_T_ values to measured values (2007–2012) by linear regression produced an r^2^ of 0.49 as the model failed to accurately predict some of the extreme low observations. However, the high values were well predicted and the overall trend and seasonal oscillations fit well with the real data (Fig 3B). The hind cast shows a decreasing trend in pH at all sites with a mean decrease of 0.1 and 0.08 pH_T_ units at site 1 and 2, respectively, since the start of the 20^th^ century (Fig 4A). Average annual temperature was 1.2°C higher in 2012 than 1910 at site 1 and 0.41°C higher in 2012 than 1921 at site 2 (Fig 4B). The calculated annual average pH at site 1 and 2 in the years of sample collection was 8.18 and 8.19 compared to 8 at Point B in 2012. Calculated annual average aragonite saturation state at site 1 and 2 during the years of sample collection was 3.97 and 3.88, respectively, in 2012, at Point B, average saturation state was 3.4 (Table 1).

**Table 1:** Details of sampling time, location and hydrography.

Species	Location	Date	Site depth	Maximum sampling depth	Mesh size(μm)	Annual average temperature (°C)	Annual average pH_T_	Annual Ω_a_(1 m depth)
*S*. *subula*	Site 1: 37.15° N, 2.55°E	27/09/1921	~ 2750 m*	250 m	200	17.68	8.19	3.88
*C*. *inflexa*	Site 2: 43.54° N, 7.19°E	09/07/1910	1082 m	300 m	N/A	18.55	8.18	3.97
*C*. *inflexa*	Point B: 43.41°N, 7.19°E	01/01/2012–31/03/2012	200 m	70 m	680	18.85	8.09	3.4
*S*. *subula*	Point B: 43.41°N, 7.19°E	01/08/2012–31/08/2012	200 m	70 m	680	18.85	8.09	3.4

* Depth not provided in cruise report found by mapping the coordinates on the European Marine Observation and Data Network’s Portal for Bathymetry (http://portal.emodnet-bathymetry.eu/mean-depth-full-coverage).

### Ontogenetic development

**Fig 5:**
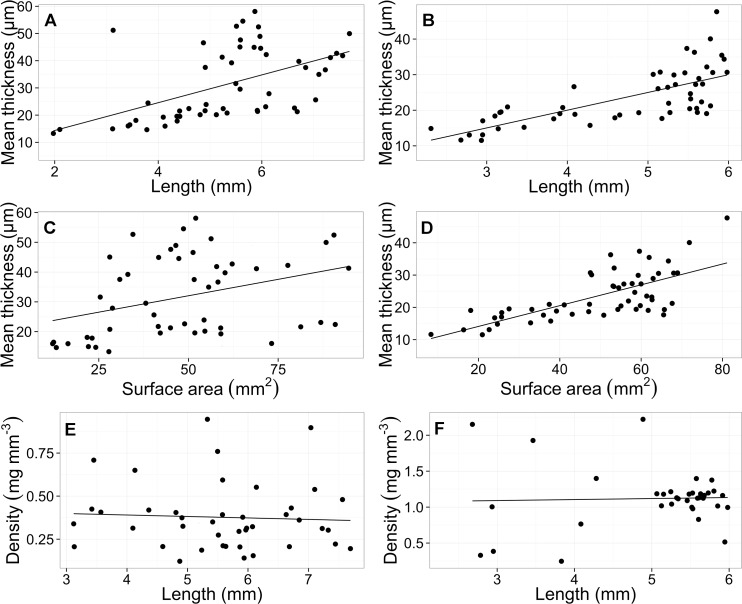
Biometric correlations of modern samples of *S*. *subula* and *C*. *inflexa* show positive correlations between average shell thickness (μm^3^) and (A) shell length (mm) and (B) shell surface area (mm^2^). (C) Shell density (mg mm^-3^) plotted against shell length (mm) shows no correlation.

Biometric correlations plotted for the modern samples indicate that, for both species, shell thickness increases linearly with shell length (*C*. *inflexa*: y = 0.09 + 4.9 x R^2^ 0.49, *S*. *subula*: y = 4.14 + 5.11 x R^2^ 0.26 Fig 5A) and surface area (*C*. *inflexa*: y = 7.7 + 0.32 x R^2^ 0.49, *S*. *subula*: y = 21.08 + 0.22 x R^2^ 0.11; Fig 5B); although, as observed by Bé et al. [48], there is higher variability in average shell thickness at larger shell surface areas and lengths. Shell density is uncorrelated to shell length (*C*. *inflexa*: y = 1.05 + 0.01 x R^2^–0.03, *S*. *subula*: y = 0.73 + -0.06 x R^2^ 0.03; Fig 5C).

### Shell properties

**Fig 6:**
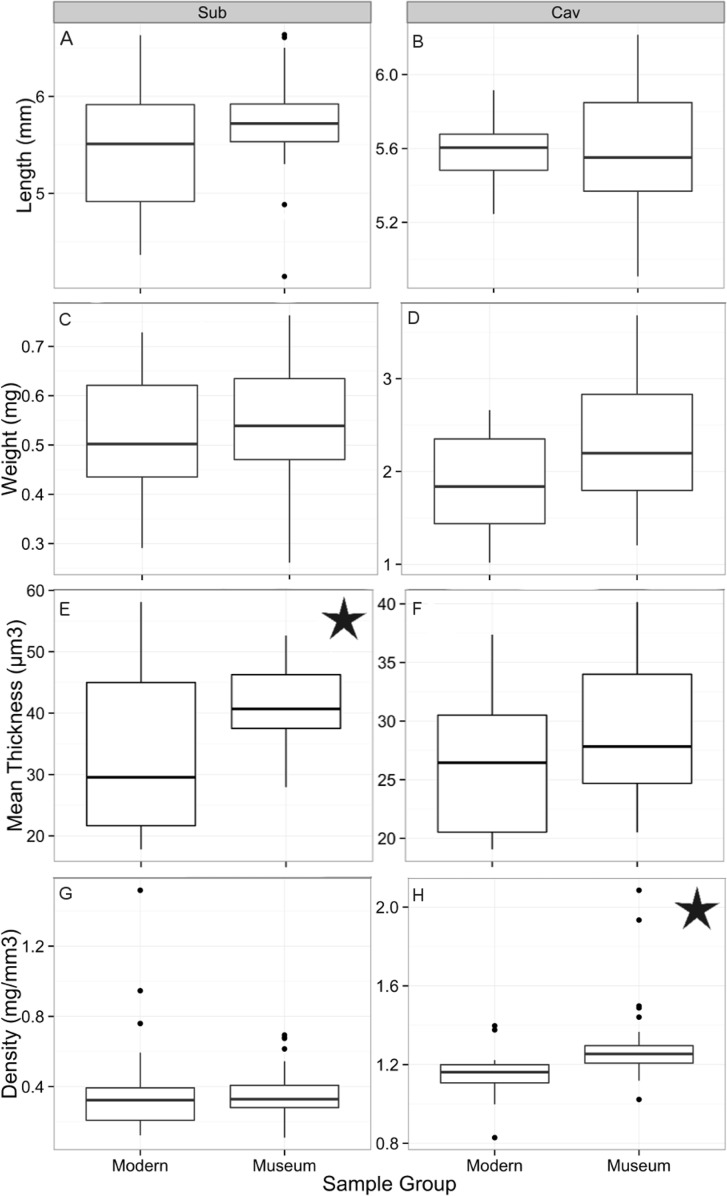
Comparison of the average shell length (CT scan; A, B) weight (manual measurements; C, D) thickness (CT scan; E, F) and density (calculated; G, H) modern 2012 and historical samples (1910/1921) of *C*. *inflexa* (Cav) and *S*. *subula* (Sub) of the same size range. Stars denote a statistically significant difference between modern and historical samples.

**Fig 7:**
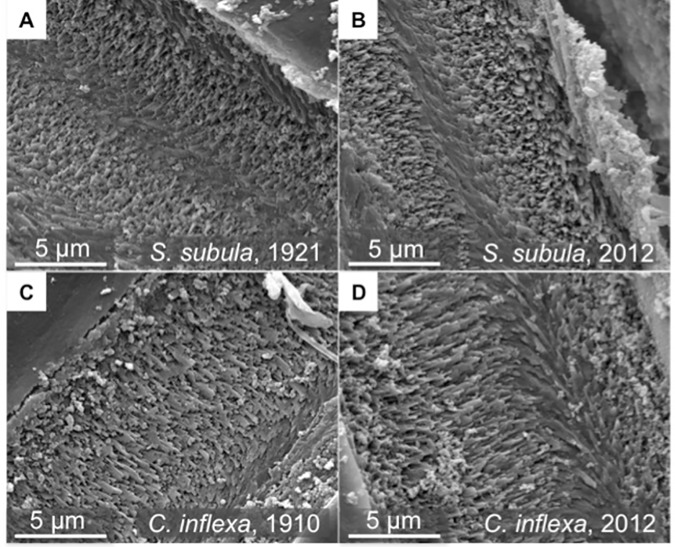
SEM micrographs of embedded and polished cross sections of 1921 (A) and 2012 (B) *S*. *subula* shells and 1910 (C) and 2012 (D) *C*. *inflexa* shells. No difference between the organisation or packing of the aragonite nanofibers can be observed in either species despite significantly higher density of historical samples of *C*. *inflexa*.

**Fig 8:**
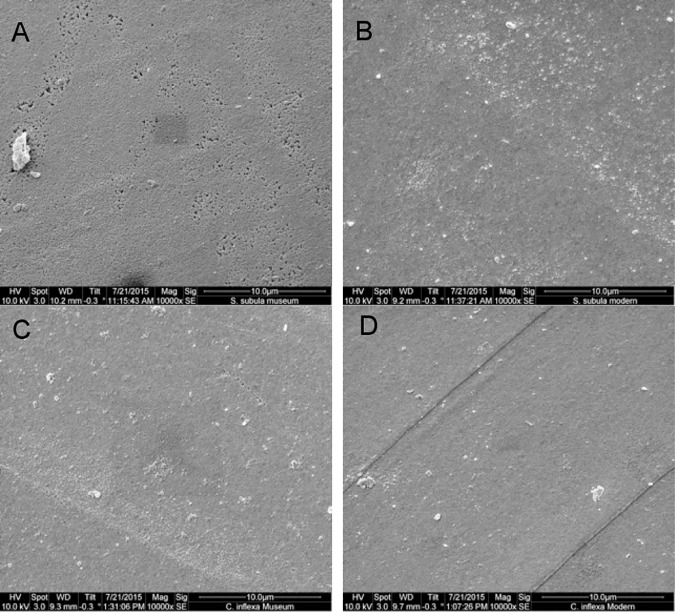
SEM micrographs of the exterior surface of *S*. *subula* shells from 1921 (A) and 2012 (B). Samples from 1921 exhibit the first signs of Type I dissolution with increased porosity. The exterior shell surface of *C*. *inflexa* shells from 1910 (C) and 2012 (D), no evidence of dissolution is present in either.

A size filter based on the maximum and minimum lengths and widths of the historical samples was applied to the modern samples to compare them to historical shells of the same size range (Fig 6A and 6B). There was no significant difference between the shell lengths of the historical (n = 27) and modern (n = 31 after size filtering) samples of *S*. *subula* as determined by CT scan (W = 540, p = 0.06; Fig 6A), nor was there any significant difference in shell weight (Fig 6C). The mean thickness of *S*. *subula* samples from 1921 (mean = 40.6 μm ± 12.7 μm) was significantly greater (W = 550, p = 0.04; Fig 5E) than that of the 2012 samples (mean = 30.59 μm ± 27.5 μm; Fig 6E), however, there was no significant difference between the shell densities of historical and modern samples (Fig 6G). Cross sections of a subset of *S*. *subula* shells showed the characteristic interlocked, helical, aragonite nanofibers in both species and no discernable differences in crystal structure or degradation between the old and new samples (Fig 7A and 7B). Inspection of the outer surface of the shells using SEM revealed shells to be in good condition with no evidence of major dissolution. Closer inspection found no dissolution in any of the subset of 10 modern samples of *S*. *subula*; however, all 10 historical samples exhibited slightly increased porosity in some places, possibly the effects of long term storage (Fig 8A and 8B).

There was no significant difference between the shell lengths of the historical (n = 30) and modern (n = 18 after size filtering) samples of *C*. *inflexa* (W = 315, p = 0.89; Fig 6B), nor in shell weight (Fig 6D). Samples of *C*. *inflexa* from 1910 were significantly denser than 2012 samples (W = 567, p = 0.002; mean 1910 samples = 1.3 ± 0.68, mean 2012 samples = 1.1 ± 0.78; Fig 6H) but there was no significant difference between the average shell thickness of the historical and modern *C*. *inflexa* samples (Fig 6F). As for *S*. *subula*, aragonite nanofibers were helically arranged and no differences in organization or packing of the crystals were observed between the old and new samples (Fig 7C and 7D). Inspection of the outer surface of the shells using SEM revealed shells to be in good condition with no evidence of major dissolution. Closer inspection found no dissolution in any of the 10 modern samples of *C*. *inflexa*. The historical samples were also in good condition, with the occasional, very small area of increased porosity (Fig 8C and 8D).

**Fig 9:**
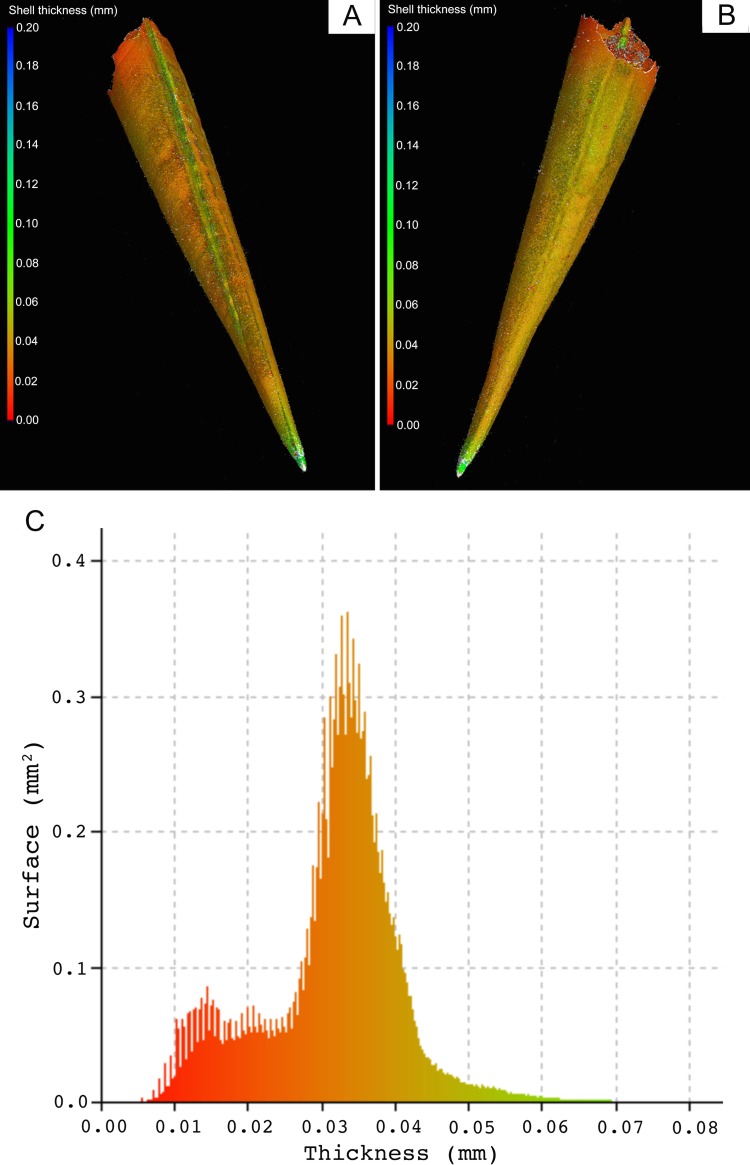
Variation in shell thickness in a *Styliola subula* individual. A and B are the same individual but with reversed orientation. The overall patterns of shell thickness in this individual are representative of what we found in all specimens. Colouring represents shell thickness at a particular point on a scale given to the left of the panel. Clearly visible are localized areas of higher thickness at the tip of the shell and along a ridge running the length of the shell. Additionally the shell tapers off to areas of much lower thickness around the opening. C; illustrates the distribution of shell thickness over the surface area of the whole shell. It is also generated by the VGStudio Max software and uses the same colour coding as used in panel A and B. It indicates that thicker areas over 0.05 mm are a minority of the shell (per unit of surface area), the majority of the shell is between 0.025 and 0.045 mm thick, but there is a significant minority of the shell is less than 0.025 mm thick.

**Fig 10:**
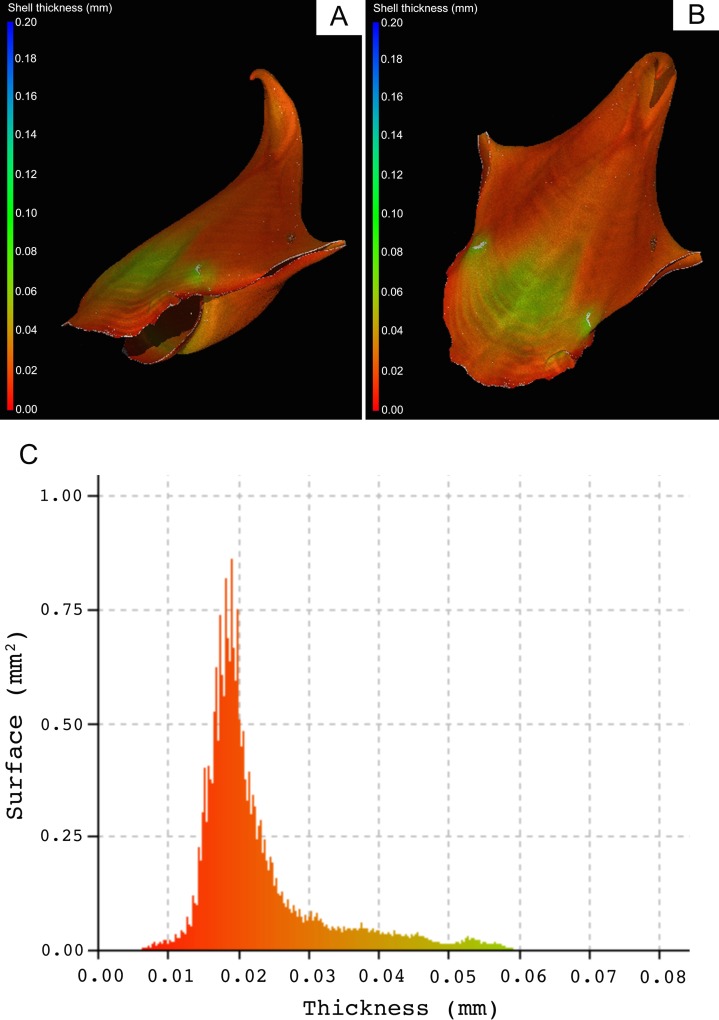
Variation in shell thickness in a *Cavolinia inflexa* individual. A and B are the same individual but with different orientations The overall patterns of shell thickness in this individual are representative of what we found in all specimens. There are clearly areas of significantly different shell thickness and the localized areas of high thickness presumably represent areas where increased resistance to mechanical breakage is required. C; illustrates the distribution of shell thickness over the surface area of the whole shell. It is also generated by the VGStudio Max software and uses the same colour coding as used in panel A and B. A greater area of the shell is seen to be over 0.045 mm (per unit of surface area) than observed in the *S*. *Subula* individual in Fig 10, but the majority of the shell of this individual is less than 0.025 mm thick.

Scan results revealed a high variability in shell thickness throughout the shells of both species. In *S*. *subula* shells, the same the patterns of shell thickness are found in all specimens. Localized areas of higher thickness are present at the tip of the shell and along a ridge running the length of the shell; thickness tapers off towards the aperture (Fig 9A and 9B). All specimens of *C*. *inflexa* also show the same patterns of shell thickness. In this species, the shell is thicker near the aperture, where the top and dorsal and ventral halves of the shell meet. A thicker area is present at the front centre of the ventral side and on the dorsal ridge, running across the shell, just behind the aperture (Fig 10A and 10B).

## Discussion

In order to assess whether climate change drivers affect a certain parameter, it is important to know the temperature and pH that the animal has experienced. Historically, temperature is well recorded; however, as ocean acidification is a relatively novel field of research, there is a paucity of long term pH and carbonate chemistry data, making it necessary to estimate historical conditions. The saturation state and pH estimate produced by the hindcast show a steady decrease in the western Mediterranean since the turn of the 20^th^ century at a rate of approximately 0.008 pH units per decade at site 1 and 0.007 units per decade at site 2. This is comparable with rates reported by Touratier and Goyet [9] (pre-industrial—present day) of 0.01–0.003 pH units per decade. The effects of biological activity are not considered in the pH estimation, which may also have implications for predicting the exact pH. Records of atmospheric CO_2_ are better documented, through the long-term time series at Mauna Loa, however values from ice core data must be used to attain atmospheric pCO_2 atm_ prior to the start of the series in 1958. Temperature and salinity records are more readily available, although with many gaps over the last hundred years. Due to missing salinity data, the values for the pH hindcast were extrapolated backwards from a time series ending in 1957. To assess the relevance of these time series data, the values were compared to the *in-situ* measurements of temperature and salinity taken on the Dana and Thor cruises and found to be within the range of the temperature and salinity data collected at the same time as the historical pteropod samples (salinity ± 0.5, temperature ± 0.3°C) [37, 38]. A major disadvantage of this technique is that the hindcast is for surface waters (1 m depth); attempts to estimate pH for 50 m produced unacceptably high errors when compared with real measurements. Due to the short residence time of Mediterranean waters, it has been estimated that the entire water column has been contaminated with anthropogenic CO_2_. Touratier and Goyet [9] also show that, in the area surrounding Point B, anthropogenic CO_2_ is homogeneously distributed over the first 500 m water depth, thus the rate of change of pH_T_ due to ocean acidification should be representative of the net sampling depth for all samples (≤ 300 m).

The novel approach of using nano-CT scanning to assess shell properties offers the advantage of yielding many different parameters per single scan. It also allows a holistic look at differences in the entire shell, not just small sections, giving an idea of the effects over the lifetime of the animal and taking into account areas of differing thickness due to shell structure morphology. Scanned samples showed a wide variation in wall thickness in different parts of the shell, particularly in *C*. *inflexa* (Figs 9 and 10). Until now, it has been unclear that such variation existed and has implications for studies of shell thickness that use measurements taken at a single point on the shell. In future biometric work, there is the potential to build on these insights from CT scanning and investigate localized and zonal differences in shell thickness and dimensions. For example CT data could potentially be used to probe changes in the higher thickness and lower thickness areas of the shell indicated in Figs 9 and 10 in isolation from each other.

If CT scanning of pteropod shells is to be employed as a method of assessing global change, it is necessary to select standard shell parameters to analyse. The ontogenetic analysis was undertaken to assess which parameters were unaffected by different life stages. The work of Roger et al., [30], who used a different technique to compare thickness of *Diacavolinia longirostris* and *Creseis clava* from samples ranging from 1960–2000,found significant decreases in thickness in modern *D*. *longirostris* compared to older samples. Decreases in shell thickness in response to ocean acidification have been reasonably well documented in other mollusc species [49–51]; however, the comparison for pteropods is not as straight forward due to the shell growth phases. Studies of the shells of *Cuvierina columnella* indicate that Cavoliniidae shell growth occurs in two phases; during phase 1 the shell grows to its maximum size and then, in phase 2, continues to thicken throughout the rest of the animal’s life [48]. To attempt to account for the continuous thickening, a wide range of modern samples (n = 59 for *C*. *inflexa*, n = 57 for *S*. *subula)* were measured to compare the offset between thicknesses at the full range of shell lengths. Increased variability of shell thickness is observed as shell length increases (Fig 5A), so this metric should be treated with caution if used as the sole parameter for monitoring climate change effects. Corals have been observed to produce denser aragonite under higher pH conditions with more tightly packed and well-formed crystals compared to those accreted under lower pH conditions [52, 53], for this reason, density was also assessed as a potential metric. Density was uncorrelated to shell length and, thus, appears to be unaffected by ontogenetic stage (Fig 5C). For the purposes of this study, density was calculated from measured values; this method is not ideal as accuracy may be compromised via propagation of errors. In theory, density could be measured using the CT scanner, making this metric much more attractive for use in monitoring studies.

To test the application of these monitoring metrics, a comparison was made between historical and modern samples of *C*. *inflexa* and *S*. *subula*. As pteropods are notoriously difficult to maintain in laboratory cultures [54], it was not possible to use a perturbation experiment to assess any differences in shell morphometrics via CT scanning; there would have been too little shell produced during the amount of time that it is viable to maintain healthy cultures. The use of historical samples enabled the application of CT scanning over the whole shell (as opposed to a small section grown under culture conditions) and also provided a rare opportunity to tentatively assess any evidence of long term effects of climate change. The use of historical samples does have associated issues, the samples only provide a “snapshot” of the collection conditions and measurements of environmental parameters taken at the time can be difficult to locate, incomplete or missing entirely. This makes it challenging to attribute observed differences to any one parameter with confidence. There is also the issue of the treatment and storage of historical samples, even the best-kept samples may be subject to minor damage over the course of several decades, and long term storage may produce dissolution effects. *Styliola subula* samples used in this study exhibited increased porosity compared to modern specimens; one of the first signs of type I dissolution as characterised by Bednaršek et al. [27], possibly due to their prolonged storage in ethanol.

Despite the maintenance of super saturated conditions, differences were observed in shell thickness and density. Historical samples of *S*. *subula* shells were significantly thicker than modern shells. Maximum shell length of adult *S*. *subula* is 13 mm [55]; the *S*. *subula* included in the study are far below maximum size and the modern samples were collected during the same months as the 1921 samples (Table 1). Assuming no significant changes in breeding season, these factors minimise the likelihood of differences in shell thickness being related to ontogenetic effects and more likely that they are caused by environmental drivers. No significant difference was observed in the shell thickness of historical and modern *C*. *inflexa*. The *C*. *inflexa* shells are much closer to their recorded maximum length of 8 mm [55] and it was not possible to collect samples from the same month of the year so ontogentic effects cannot be excluded. It is unclear exactly how long pteropods live for but the consensus in the literature is that they have a lifespan of at least 1 year; therefore, the morphometric signal observed in the adult specimens would likely have been integrated over multiple seasons, minimising year on year variability.

Despite no significant difference in the weight, or thickness of modern and historical samples, *C*. *inflexa* shells from 1910 were significantly denser than modern shells. A closer inspection of the results reveals that, although there was no statistically significant difference in these parameters, the historical samples of *C*. *inflexa* were slightly heavier and had a more variable thickness than the modern samples, which together, may account for the difference in density. *Styliola subula* displayed no significant difference in shell density between the modern and historical samples. It seems unlikely that pH conditions are the reason why density differences are seen in one species and not the other. The annual average pH and saturation states for the years of historical sample collection are very similar for the two sites, pH only differing by 0.01 units and Ω_a_ by 0.09 units (Table 1). It is possible that the increased porosity observed on the outside of the historical samples of *S*. *subula* could be the reason why no significant difference in density was observed between the historical and modern samples. The samples also came from different locations so the effect of local environmental conditions, such as food availability or another factor resulting in reduced resource or ability to calcify, may be the cause. Unlike previous studies investigating the effects of pH on the ultrastructure of corals, it was not possible to observe any change in the packing or morphology of the aragonite rods in either species, despite there being a statistically significant change in density in one species. This could be due to the fact that the saturation states experienced by our specimens are much higher than those used in laboratory based perturbation experiments with corals [52].

As long term studies of the effects of climate change drivers are very scare, the analysis of historical samples could provide a rare view on the long term effects of climate change. Any such analysis should be viewed tentatively, taking into account the aforementioned limitations of using a small sample size of historical samples from different locations. Despite the high level of uncertainty, our results demonstrate empirical differences between the shell thickness and density of historical and modern samples of two species of Mediterranean pteropods. Dissolution during long-term storage of historical samples in ethanol could not have produced the observed trends as historical specimens were found to be thicker. Caution should be exercised in attributing the changes to either a temperature or pH effect as a wide variety of local drivers, such as dissolved inorganic carbon, salinity, and resource availability could also have affected shell properties. Geochemical comparisons of modern and historical samples could be used to clarify the hydrological conditions experienced during the lifetime of the animal but this was outside of the scope of this study.

The results are in agreement with experimental work indicating that decreasing pH (or Ω_a_) and increasing temperature reduce calcification [22, 24, 25] and are also supported by the work on historical samples carried out by Roger et al. [30]. It is unclear whether the observed effects are due to solely pH or Ω_a_ change or an interactive effect with temperature as no studies on Mediterranean species have investigated the effects of temperature on calcification rate. The changes in density have not caused any change to the aragonite ultrastructure but work with corals has demonstrated structural changes to aragonite at saturation states as high as 2.28 [53]. At the projected levels of CO_2 atm_ for the year 2100 [56] a reduction in Ω_a_ to around these values is predicted for the Mediterranean. The complex structures of pteropods shells give it flexibility and hardness whilst maintaining a light weight [57], and any ultra-structural changes may compromise these properties. The suggestion that pteropod shells are getting thinner in synchrony with changes to temperature and saturation state indicates that there may be an increased risk of predation pressure on Mediterranean pteropods. Although long-term decreases have not yet been observed in pteropod populations in the north west Mediterranean [18], the observed changes to shell thickness and density could indicate that a tipping point may occur in the future.

More work is required to further explore the application of such a method, larger sample sizes should be used and, preferably supported by laboratory calibrations with individuals grown in controlled conditions. The high resolution of new CT scanners allows analysis of very small samples. As juveniles are often easier to incubate than adults [54], it may be viable to test the method with pteropods reared from eggs. Due to the confounding effects of other environmental parameters when using natural samples, this method is probably best employed in conjunction with other techniques, such as biogeochemical analysis, for assessing climate change drivers. It is also worth noting that the CT-scanning approach is one of the first to visualize and quantify the variability in shell thickness, so that these uncertainties can be better appreciated. Follow up work could attempt to separate data into thickness “domains” to look for thinning of specific regions of the shell; however with current data analyses approaches, this would be a time consuming task and was outside the scope of this current study. Despite some uncertainties, the differences in density and thickness between historical and modern samples of two separate species, collected at different times and locations is an encouraging indication of the potential for using micro CT scan methods one of a suite of tools for assessing impacts of ocean acidification in supersaturated basins.

## Supporting Information

S1 Fig Screenshot of initial 3D reconstruction of a typical CT scan in VG StudioMax software. Each scan comprised two pteropod shells (one modern, one museum sample) and a calcite rhomb standard. The samples were encased in a balsa wood mount but as you can see by excluding low X-ray attenuating materials from the 3D reconstruction (the abundant lower intensity values in the histogram in the panel to the right) this mount is effectively removed.
(DOCX)

S2 Fig Objects in the initial reconstruction are separated into three distinct files using a process called 3-2-1 registration. (DOCX)

S3 Fig Individual objects are now ready for dimension, thickness, and volume analyses. This image shows how organic material (light grey) inside the shell are excluded from the volume and thickness analyses of the shell (colored) through selection of greyscale boundaries to be considered. We attempted to keep the boundaries where this selection was carried out constant between analyses. In some cases this was not possible and image specific grey scale boundaries were necessary, and this is an accepted potential source of error in our treatment. It is an area that could potentially be improved upon in future studies through a specialized software to identify different materials based on absorption criteria, however we feel it is somewhat out of the scope of this initial study. CT scanning software used proprietary algorithms to calculate parameters such as thickness, volume etc. Therefore it is not possible to give an in depth description of how the software calculates this. Broadly speaking though the three dimensional unit is known as a “voxel”. Therefore thickness and volume of the 3D CT scanner rendering represent the distance at a certain point in voxel units, or the total volume in voxels occupied. Each CT scan will have different dimensions of its voxels depending on its resolution, so the size of the voxel is not constant between scans.
(DOCX)

S4 Fig In some cases extraneous objects in the mount were not removable based on greyscale intensity differences, for example see the “floating material above and below the shell in the top panel. In the top panel you can see that they are colored, which means that they have erroneously been included in the wall-thickness analyses that may bias the results. However they could be removed by component analyses where the continuous shell is selected as a single component (colored, bottom panel) and all other objects (grey) are excluded.
(DOCX)

S5 Fig Shell length (A, B) as derived via measurements made by CT scan plotted against shell length as derived by measuring using a binocular microscope for all samples of *S*. *subula* (Sub) and *C*. *inflexa* (Cav). Solid grey line represents the linear regression between the two groups, dashed line represents a 1:1 regression, which would equate to exactly the same values derived by both measurements techniques. (C, D) As above, for shell width. Reproducibility was assessed by replica analysis of the carbonate rhomb standard and an individual *S*. *subula* specimen that was analysed multiple times. CT based measurements were compared to measurements taken with a light microscope, which gives an opportunity to assess potential biases in both approaches. Individual standard analyses is given are S1 and S2 Tables. Average values for the petropod standard +/- one standard deviation are from n = 15 analyses. Average thickness (**μm**): 40.8 +/- 3.6, volume (mm): 0.943 +/- 0.176: Surface area (mm): 53.702 +/- 10.1. The data indicate that for these samples the surface area determination is the least precise and the volume the most precise. Calcite rhomb data was used to assess the precision of dimension measurements in particular. Average standard data from n = 28 analyses. Average Volume = 4.12 +/- 0.07. Dimension 1: 2.00 +/- 0.05, Dimension 2: 1.99 +/- 0.05.
(DOCX)

S1 Table Time series of measured water temperature, salinity and corbonate chemistry parameters taken from Point B from 2007–2012, used to validate hindcast modeling of pH. (DOCX)

S2 Table Modern specimens used in analysis of shell morphometrics based on ontogenetic stage. (DOCX)

S3 Table The modern and museum specimens used for comparison. A size filter has been applied to the modern samples to select those within the same length and width range as the museum samples. “New” denotes samples from 2012 and “Old” denotes museum samples.
(DOCX)
