# Supplementary material for: Comparison of Mediterranean Pteropod Shell Biometrics and Ultrastructure from Historical (1910 and 1921) and Present Day (2012) Samples Provides Baseline for Monitoring Effects of Global Change
Source: PLoS One. 2017 Jan 26;12(1):e0167891. doi: 10.1371/journal.pone.0167891 (PMC5268398; doi:10.1371/journal.pone.0167891)
Supplement: S1 Fig — Each scan comprised two pteropod shells (one modern, one museum sample) and a calcite rhomb standard. The samples were encased in a balsa wood mount but as you can see by excluding low X-ray attenuating materials from the 3D reconstruction (the abundant lower intensity values in the histogram in the panel to the right) this mount is effectively removed. (DOCX) [file pone.0167891.s001.docx]

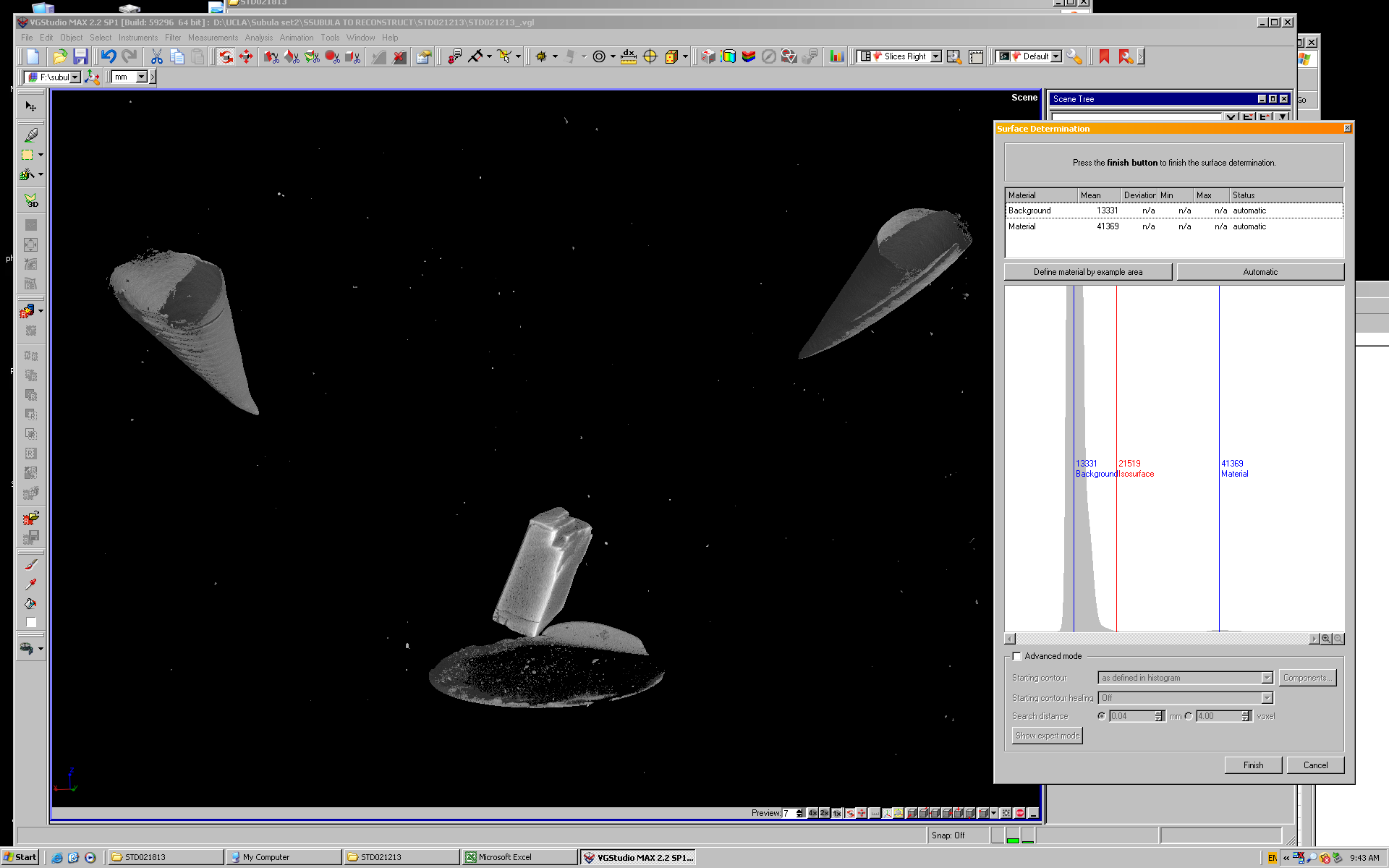


S1 Fig: Screenshot of initial 3D reconstruction of a typical CT scan in VG StudioMax software. Each scan comprised two pteropod shells (one modern, one museum sample) and a calcite rhomb standard. The samples were encased in a balsa wood mount but as you can see by excluding low X-ray attenuating materials from the 3D reconstruction (the abundant lower intensity values in the histogram in the panel to the right) this mount is effectively removed.
