# Supplementary material for: Comparison of Mediterranean Pteropod Shell Biometrics and Ultrastructure from Historical (1910 and 1921) and Present Day (2012) Samples Provides Baseline for Monitoring Effects of Global Change
Source: PLoS One. 2017 Jan 26;12(1):e0167891. doi: 10.1371/journal.pone.0167891 (PMC5268398; doi:10.1371/journal.pone.0167891)
Supplement: S2 Fig — (DOCX) [file pone.0167891.s002.docx]

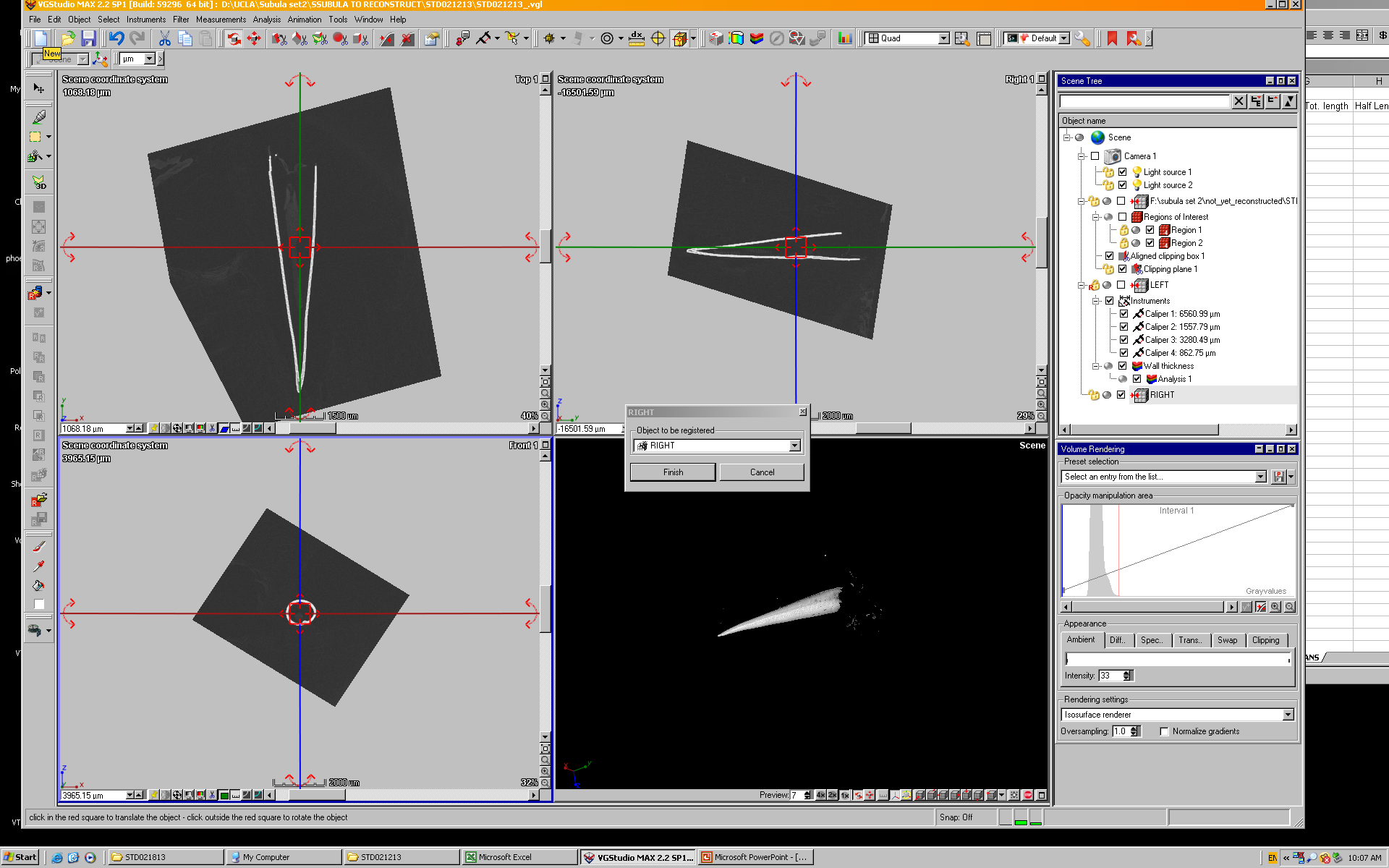


S2 Fig: Objects in the initial reconstruction are separated into three distinct files using a process called 3-2-1 registration.
