# Supplementary material for: Comparison of Mediterranean Pteropod Shell Biometrics and Ultrastructure from Historical (1910 and 1921) and Present Day (2012) Samples Provides Baseline for Monitoring Effects of Global Change
Source: PLoS One. 2017 Jan 26;12(1):e0167891. doi: 10.1371/journal.pone.0167891 (PMC5268398; doi:10.1371/journal.pone.0167891)
Supplement: S3 Fig — This image shows how organic material (light grey) inside the shell are excluded from the volume and thickness analyses of the shell (colored) through selection of greyscale boundaries to be considered. We attempted to keep the boundaries where this selection was carried out constant between analyses. In some cases this was not possible and image specific grey scale boundaries were necessary, and this is an accepted potential source of error in our treatment. It is an area that could potentially be improved upon in future studies through a specialized software to identify different materials based on absorption criteria, however we feel it is somewhat out of the scope of this initial study. CT scanning software used proprietary algorithms to calculate parameters such as thickness, volume etc. Therefore it is not possible to give an in depth description of how the software calculates this. Broadly speaking though the three dimensional unit is known as a “voxel”. Therefore thickness and volume of the 3D CT scanner rendering represent the distance at a certain point in voxel units, or the total volume in voxels occupied. Each CT scan will have different dimensions of its voxels depending on its resolution, so the size of the voxel is not constant between scans. (DOCX) [file pone.0167891.s003.docx]

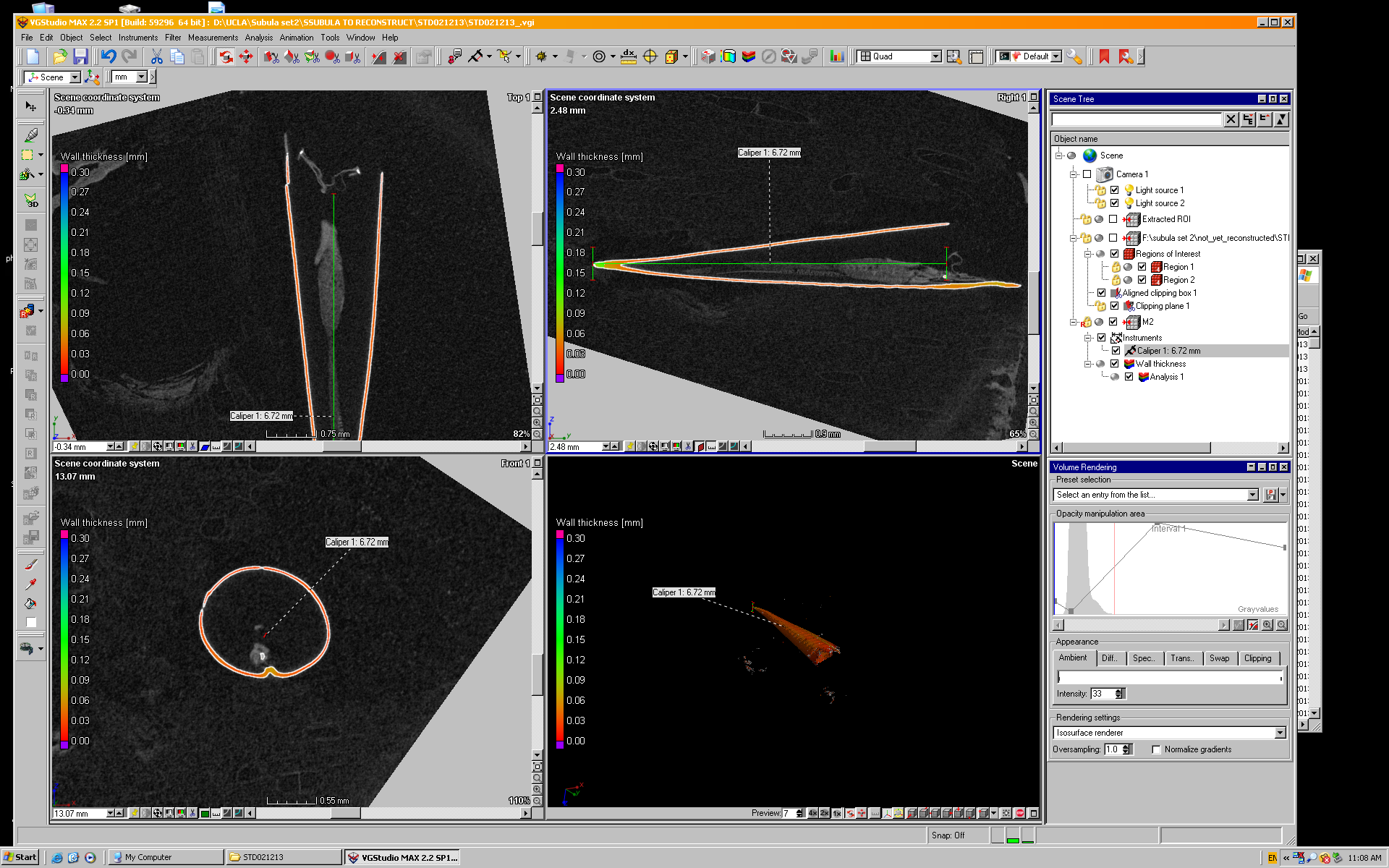


S3 Fig: Individual objects are now ready for dimension, thickness, and volume analyses. This image shows how organic material (light grey) inside the shell are excluded from the volume and thickness analyses of the shell (colored) through selection of greyscale boundaries to be considered. We attempted to keep the boundaries where this selection was carried out constant between analyses. In some cases this was not possible and image specific grey scale boundaries were necessary, and this is an accepted potential source of error in our treatment. It is an area that could potentially be improved upon in future studies through a specialized software to identify different materials based on absorption criteria, however we feel it is somewhat out of the scope of this initial study. CT scanning software used proprietary algorithms to calculate parameters such as thickness, volume etc. Therefore it is not possible to give an in depth description of how the software calculates this. Broadly speaking though the three dimensional unit is known as a “voxel”. Therefore thickness and volume of the 3D CT scanner rendering represent the distance at a certain point in voxel units, or the total volume in voxels occupied. Each CT scan will have different dimensions of its voxels depending on its resolution, so the size of the voxel is not constant between scans.
