# Supplementary material for: Comparison of Mediterranean Pteropod Shell Biometrics and Ultrastructure from Historical (1910 and 1921) and Present Day (2012) Samples Provides Baseline for Monitoring Effects of Global Change
Source: PLoS One. 2017 Jan 26;12(1):e0167891. doi: 10.1371/journal.pone.0167891 (PMC5268398; doi:10.1371/journal.pone.0167891)
Supplement: S4 Fig — In the top panel you can see that they are colored, which means that they have erroneously been included in the wall-thickness analyses that may bias the results. However they could be removed by component analyses where the continuous shell is selected as a single component (colored, bottom panel) and all other objects (grey) are excluded. (DOCX) [file pone.0167891.s004.docx]

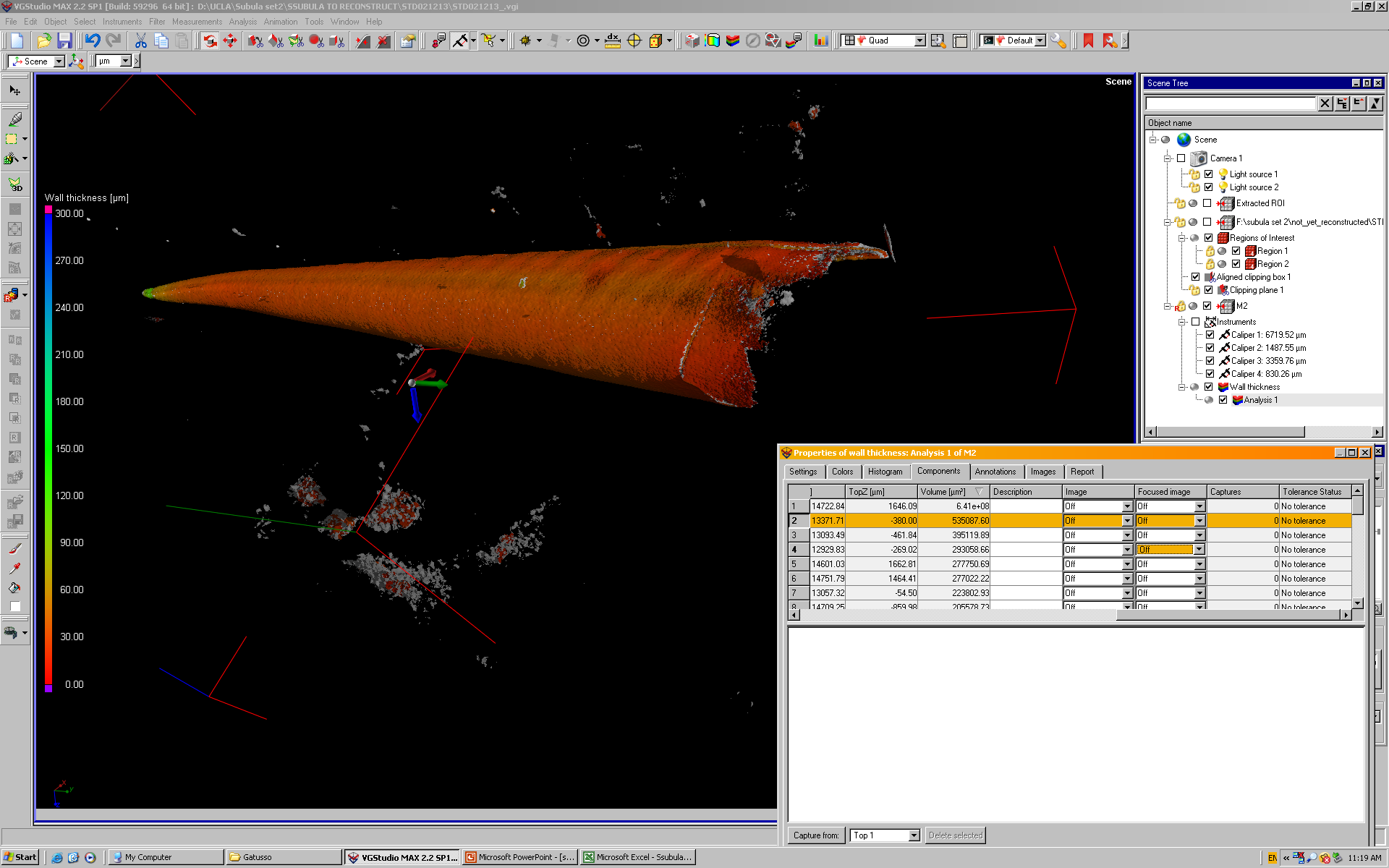


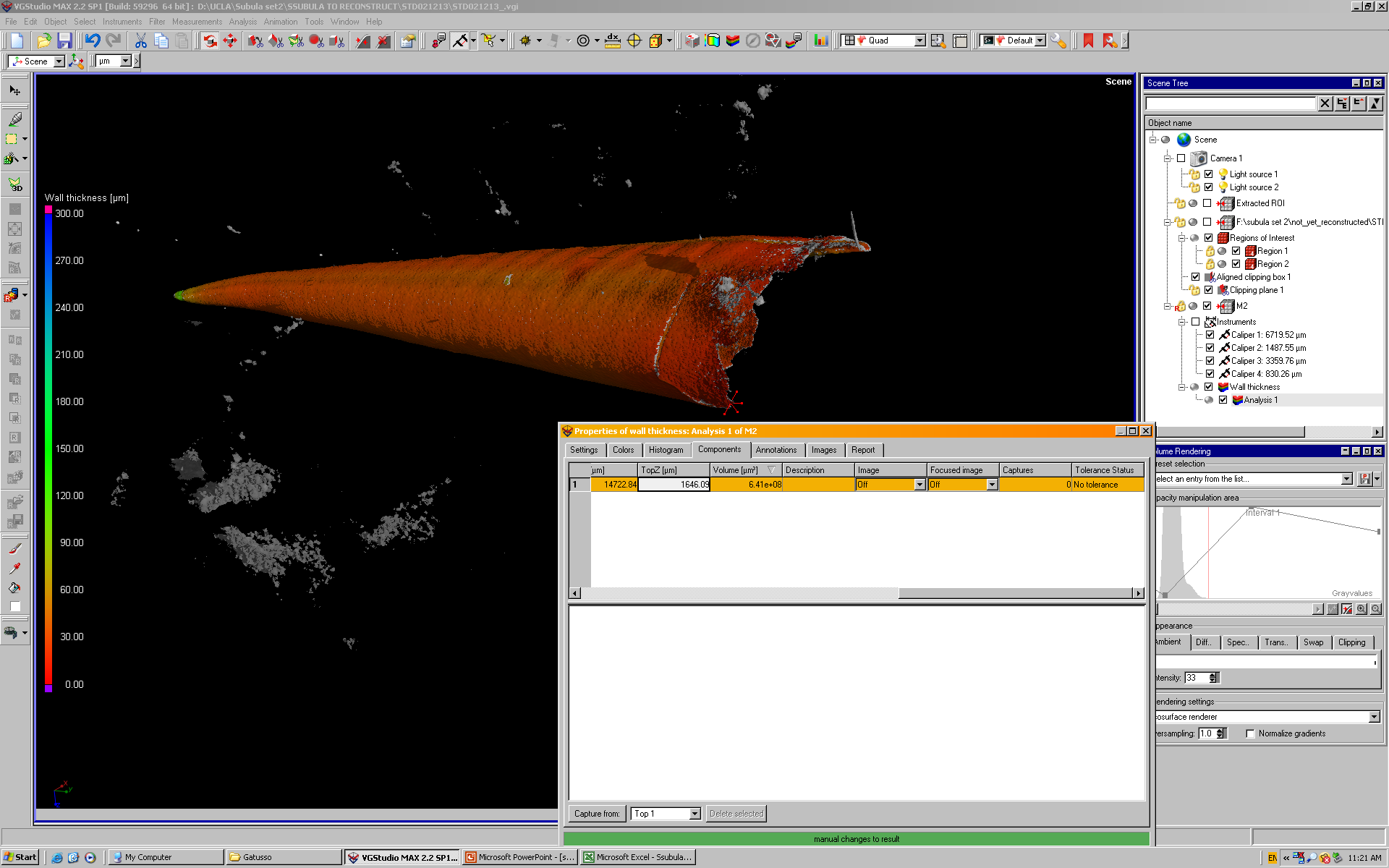


S4 Fig: In some cases extraneous objects in the mount were not removable based on greyscale intensity differences, for example see the “floating material above and below the shell in the top panel. In the top panel you can see that they are colored, which means that they have erroneously been included in the wall-thickness analyses that may bias the results. However they could be removed by component analyses where the continuous shell is selected as a single component (colored, bottom panel) and all other objects (grey) are excluded.
