# Supplementary material for: Comparison of Mediterranean Pteropod Shell Biometrics and Ultrastructure from Historical (1910 and 1921) and Present Day (2012) Samples Provides Baseline for Monitoring Effects of Global Change
Source: PLoS One. 2017 Jan 26;12(1):e0167891. doi: 10.1371/journal.pone.0167891 (PMC5268398; doi:10.1371/journal.pone.0167891)
Supplement: S5 Fig — Shell length (A, B) as derived via measurements made by CT scan plotted against shell length as derived by measuring using a binocular microscope for all samples of S. subula (Sub) and C. inflexa (Cav). Solid grey line represents the linear regression between the two groups, dashed line represents a 1:1 regression, which would equate to exactly the same values derived by both measurements techniques. (C, D) As above, for shell width. Reproducibility was assessed by replica analysis of the carbonate rhomb standard and an individual S. subula specimen that was analysed multiple times. CT based measurements were compared to measurements taken with a light microscope, which gives an opportunity to assess potential biases in both approaches. Individual standard analyses is given are S1 and S2 Tables. Average values for the petropod standard +/- one standard deviation are from n = 15 analyses. Average thickness (μm): 40.8 +/- 3.6, volume (mm): 0.943 +/- 0.176: Surface area (mm): 53.702 +/- 10.1. The data indicate that for these samples the surface area determination is the least precise and the volume the most precise. Calcite rhomb data was used to assess the precision of dimension measurements in particular. Average standard data from n = 28 analyses. Average Volume = 4.12 +/- 0.07. Dimension 1: 2.00 +/- 0.05, Dimension 2: 1.99 +/- 0.05. (DOCX) [file pone.0167891.s005.docx]

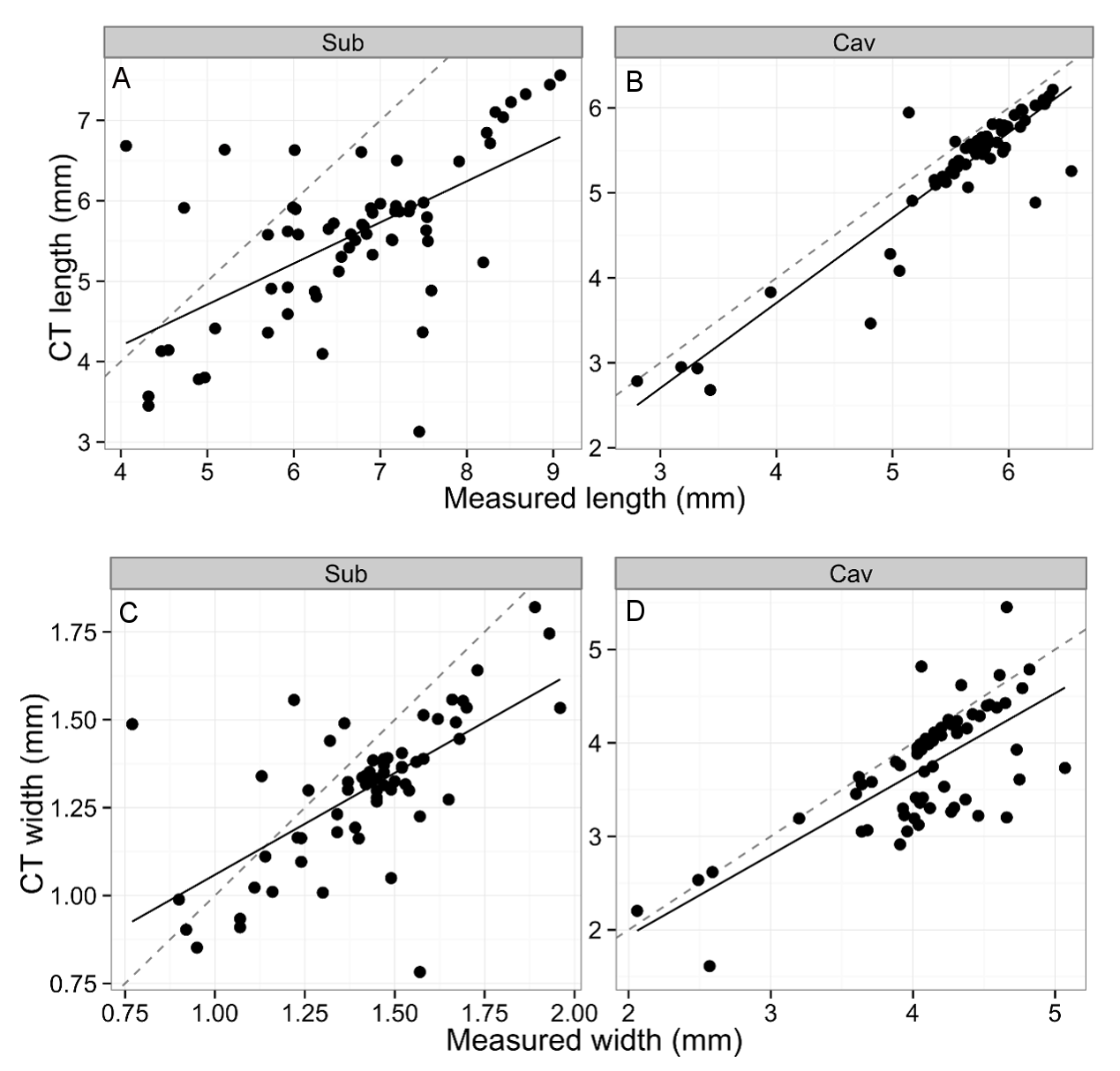


S5 Fig: Shell length (A, B) as derived via measurements made by CT scan plotted against shell  length as derived by measuring using a binocular microscope for all samples of *S. subula* (Sub) and *C. inflexa* (Cav). Solid grey line represents the linear regression between the two groups, dashed  line represents a 1:1 regression, which would equate to exactly the same values derived by both  measurements techniques. (C, D) As above, for shell width.

Reproducibility was assessed by replica analysis of the carbonate rhomb standard and an individual *S. subula* specimen that was analysed multiple times. CT based measurements were compared to measurements taken with a light microscope, which gives an opportunity to assess potential biases in both approaches. Individual standard analyses is given are supplementary tables. Average values for the petropod standard +/- one standard deviation are from n = 15 analyses. Average thickness (**µm)**: 40.8 +/- 3.6, volume (mm): 0.943 +/- 0.176: Surface area (mm): 53.702 +/- 10.1. The data indicate that for these samples the surface area determination is the least precise and the volume the most precise. Calcite rhomb data was used to assess the precision of dimension measurements in particular. Average standard data from n = 28 analyses. Average Volume = 4.12 +/- 0.07. Dimension 1: 2.00 +/- 0.05, Dimension 2: 1.99 +/- 0.05.
