# Supplementary material for: Comparison of Mediterranean Pteropod Shell Biometrics and Ultrastructure from Historical (1910 and 1921) and Present Day (2012) Samples Provides Baseline for Monitoring Effects of Global Change
Source: PLoS One. 2017 Jan 26;12(1):e0167891. doi: 10.1371/journal.pone.0167891 (PMC5268398; doi:10.1371/journal.pone.0167891)
Supplement: S1 Table — (DOCX) [file pone.0167891.s006.docx]

S1 Table: Time series of measured water temperature, salinity and corbonate chemistry parameters taken from Point B from 2007-2012, used to validate hindcast modeling of pH.

| **Date** | **Salinity** | **Temperature (˚C)** | **C_T_**  **(µmol kg^-1^)** | **A_T_**  **(µmol kg^-1^)** | **Ω aragonite** |
| --- | --- | --- | --- | --- | --- |
| 09/01/07 | 38.16 | 15.46 | 2248.5 | 2561.4 | 3.32 |
| 16/01/07 | 38.21 | 15.45 | 2252.5 | 2564.8 | 3.31 |
| 23/01/07 | 38.24 | 15.42 | 2252.7 | 2565.8 | 3.32 |
| 30/01/07 | 38.18 | 14.95 | 2253 | 2560.6 | 3.26 |
| 06/02/07 | 38.17 | 14.75 | 2257.3 | 2563 | 3.24 |
| 13/02/07 | NA | NA | NA | NA | NA |
| 20/02/07 | 38.1 | 14.27 | 2253.1 | 2555.9 | 3.20 |
| 27/02/07 | NA | NA | NA | NA | NA |
| 06/03/07 | NA | NA | NA | NA | NA |
| 13/03/07 | NA | NA | NA | NA | NA |
| 20/03/07 | 38.03 | 14.24 | 2254.8 | 2554.7 | 3.18 |
| 27/03/07 | NA | NA | NA | NA | NA |
| 03/04/07 | 38.08 | 14.1 | 2252.7 | 2553.4 | 3.18 |
| 10/04/07 | NA | NA | NA | NA | NA |
| 17/04/07 | 38.01 | 15.77 | 2235.9 | 2541.6 | 3.24 |
| 24/04/07 | 37.87 | 18.04 | 2227.9 | 2537.3 | 3.32 |
| 02/05/07 | 37.72 | 18.4 | 2215.7 | 2529.8 | 3.38 |
| 09/05/07 | 37.33 | 18.82 | 2190 | 2497.6 | 3.32 |
| 15/05/07 | 37.57 | 20 | 2196.6 | 2510.5 | 3.40 |
| 22/05/07 | 37.47 | 20.45 | 2190.1 | 2510.3 | 3.47 |
| 30/05/07 | 37.82 | 17.1 | 2217.7 | 2528.9 | 3.32 |
| 05/06/07 | 37.71 | 20.23 | 2201.8 | 2517.4 | 3.42 |
| 12/06/07 | 37.56 | 21.49 | 2195.9 | 2512.4 | 3.46 |
| 19/06/07 | 37.61 | 21.69 | 2198.7 | 2519.3 | 3.50 |
| 27/06/07 | 37.75 | 19.73 | 2205 | 2523.1 | 3.44 |
| 03/07/07 | 37.77 | 22.71 | 2200.2 | 2524.6 | 3.56 |
| 10/07/07 | 37.81 | 22.1 | 2204.4 | 2522.4 | 3.48 |
| 17/07/07 | 37.88 | 22.9 | 2203.1 | 2524.5 | 3.53 |
| 24/07/07 | 37.95 | 22.59 | 2207.4 | 2534.8 | 3.58 |
| 31/07/07 | 37.95 | 24.8 | 2201.7 | 2535 | 3.69 |
| 07/08/07 | 37.89 | 25.1 | 2204.6 | 2539.3 | 3.71 |
| 14/08/07 | 37.97 | 23.45 | 2206.2 | 2542.5 | 3.69 |
| 22/08/07 | 38.07 | 23.45 | 2212.5 | 2549.3 | 3.69 |
| 28/08/07 | 38 | 24.09 | 2207 | 2544.6 | 3.72 |
| 04/09/07 | 38.06 | NA | 2210.9 | 2544.3 | NA |
| 18/09/07 | 38.18 | 22.81 | 2216 | 2552.6 | 3.67 |
| 25/09/07 | 38.17 | 22.03 | 2213.1 | 2556.8 | 3.73 |
| 02/10/07 | 38.14 | 21.25 | 2207.7 | 2549 | 3.69 |
| 09/10/07 | 38.14 | 21.33 | 2204.3 | 2548.8 | 3.72 |
| 16/10/07 | 38.13 | 20.72 | 2209.5 | 2548.4 | 3.66 |
| 23/10/07 | 38.1 | 19.17 | 2207.8 | 2546.3 | 3.62 |
| 30/10/07 | 38.09 | 20.72 | 2216.1 | 2545.9 | 3.57 |
| 06/11/07 | 38.06 | 19.18 | 2213.2 | 2545 | 3.56 |
| 13/11/07 | 38.07 | 17.64 | 2218.1 | 2544.8 | 3.48 |
| 22/11/07 | 38 | 16.63 | 2198.6 | 2516.9 | 3.37 |
| 27/11/07 | 38.12 | 16.3 | 2228.1 | 2546.4 | 3.38 |
| 04/12/07 | NA | NA | NA | NA | NA |
| 11/12/07 | NA | NA | NA | NA | NA |
| 18/12/07 | 38.1 | 15.78 | 2240 | 2551.8 | 3.31 |
| 25/12/07 | NA | NA | NA | NA | NA |
| 04/01/08 | 38.1 | NA | 2273 | 2556.4 | NA |
| 08/01/08 | 38 | NA | 2248.7 | 2551 | NA |
| 18/01/08 | 37.98 | 13.74 | 2244.1 | 2545.5 | 3.18 |
| 22/01/08 | 37.97 | 13.73 | 2243.4 | 2547 | 3.20 |
| 29/01/08 | 38.04 | 13.77 | 2248.1 | 2549.9 | 3.19 |
| 05/02/08 | 38.03 | 13.62 | 2245 | 2546.6 | 3.18 |
| 12/02/08 | 38.04 | 13.58 | 2245.1 | 2548.2 | 3.19 |
| 19/02/08 | 38.11 | 13.45 | 2250.3 | 2550.3 | 3.16 |
| 26/02/08 | 38.09 | 13.49 | 2248.9 | 2553.2 | 3.21 |
| 03/03/08 | 38.09 | 13.73 | 2255.4 | 2553.3 | 3.15 |
| 12/03/08 | 38.04 | 13.39 | 2254.3 | 2547.7 | 3.10 |
| 18/03/08 | 38.09 | 13.66 | 2256.1 | 2550.7 | 3.11 |
| 26/03/08 | 38.08 | 13.49 | 2258.8 | 2553 | 3.11 |
| 01/04/08 | 38.07 | 13.69 | 2256.8 | 2553.8 | 3.14 |
| 08/04/08 | 38.04 | 13.71 | 2257.7 | 2554.7 | 3.14 |
| 15/04/08 | 37.89 | 13.81 | 2250.8 | 2546.4 | 3.13 |
| 23/04/08 | 37.9 | 14.35 | 2250.6 | 2546.8 | 3.14 |
| 28/04/08 | 37.69 | 16.18 | 2247.1 | 2550.6 | 3.25 |
| 06/05/08 | 37.64 | 16.89 | 2249.1 | 2552.6 | 3.27 |
| 13/05/08 | 37.94 | 17.24 | 2245.2 | 2548.6 | 3.26 |
| 20/05/08 | 37.8 | 18.03 | 2238.8 | 2539 | 3.24 |
| 28/05/08 | 37.78 | 18.42 | 2246.3 | 2536.2 | 3.15 |
| 03/06/08 | 37.61 | 19.97 | 2228.4 | 2538.3 | 3.37 |
| 10/06/08 | 37.58 | 20.94 | 2219.1 | 2538.5 | 3.48 |
| 17/06/08 | 37.81 | 21.01 | 2226.5 | 2530.1 | 3.32 |
| 23/06/08 | 37.7 | 22.68 | 2211.8 | 2518.7 | 3.38 |
| 01/07/08 | 37.67 | 25.24 | 2214.3 | 2533.7 | 3.57 |
| 08/07/08 | 37.77 | 24.47 | 2215.8 | 2539.7 | 3.60 |
| 16/07/08 | 37.92 | 22.25 | 2232.6 | 2535.3 | 3.33 |
| 22/07/08 | 37.75 | 23.72 | 2224.6 | 2549.9 | 3.60 |
| 28/07/08 | 38.16 | 23.31 | 2221.3 | 2552.4 | 3.63 |
| 05/08/08 | 38.17 | 25.86 | 2210.1 | 2556.4 | 3.83 |
| 12/08/08 | NA | NA | NA | NA | NA |
| 19/08/08 | 38.12 | 23.95 | 2220.1 | 2555.9 | 3.69 |
| 26/08/08 | NA | NA | NA | NA | NA |
| 02/09/08 | 38.26 | 25.23 | 2236.6 | 2539.7 | 3.39 |
| 10/09/08 | 38.25 | 22.77 | 2223.2 | 2564.1 | 3.72 |
| 16/09/08 | NA | NA | NA | NA | NA |
| 22/09/08 | 38.32 | 22.53 | 2225.1 | 2570.2 | 3.75 |
| 29/09/08 | 38.36 | 21.59 | 2243.1 | 2574.9 | 3.61 |
| 07/10/08 | 38.28 | 19.68 | 2224.5 | 2565.4 | 3.66 |
| 14/10/08 | 38.3 | 20.29 | 2225.2 | 2567.4 | 3.68 |
| 21/10/08 | 38.36 | 21.64 | 2230.4 | 2569.3 | 3.67 |
| 28/10/08 | 38.36 | 21.64 | 2218.4 | 2569.2 | 3.79 |
| 06/11/08 | 38.12 | 18.95 | 2215.9 | 2555.1 | 3.63 |
| 10/11/08 | 38 | 18.67 | 2228.3 | 2558 | 3.54 |
| 20/11/08 | 38.12 | 18.45 | 2228.9 | 2558 | 3.52 |
| 25/11/08 | 38.09 | 17.96 | 2225 | 2556.9 | 3.54 |
| 02/12/08 | 38.07 | 16.69 | 2224.2 | 2547.5 | 3.43 |
| 09/12/08 | 38.06 | 16.26 | 2232.9 | 2552 | 3.39 |
| 17/12/08 | 37.44 | 15.21 | 2232.3 | 2539.3 | 3.27 |
| 22/12/08 | 37.62 | 15.21 | 2240.6 | 2548.2 | 3.28 |
| 30/12/08 | NA | NA | NA | NA | NA |
| 06/01/09 | 38.04 | 14.57 | 2243.2 | 2556.5 | 3.31 |
| 14/01/09 | 38.06 | 14.36 | 2256.2 | 2561.1 | 3.23 |
| 22/01/09 | 38.04 | 14.15 | 2254.2 | 2556 | 3.19 |
| 27/01/09 | 38.02 | 13.94 | 2252.1 | 2554.5 | 3.20 |
| 03/02/09 | 37.99 | 13.87 | 2247.7 | 2555 | 3.24 |
| 10/02/09 | 37.51 | 13.12 | 2250.8 | 2555.3 | 3.22 |
| 17/02/09 | 37.96 | 13.65 | 2245.8 | 2551.6 | 3.23 |
| 24/02/09 | 37.9 | 13.48 | 2249.1 | 2549.9 | 3.18 |
| 03/03/09 | 37.92 | 13.59 | 2242.7 | 2542 | 3.16 |
| 10/03/09 | 37.99 | 13.71 | 2251 | 2552.1 | 3.18 |
| 17/03/09 | 37.95333333 | 14.1 | 2247.8 | 2551.5 | 3.21 |
| 23/03/09 | 38.01 | 13.97 | 2248.4 | 2554.4 | 3.23 |
| 31/03/09 | 38 | 13.8 | 2265.9 | 2547.2 | 2.99 |
| 07/04/09 | 37.59 | 15.15 | 2237.8 | 2542.8 | 3.25 |
| 14/04/09 | NA | NA | NA | NA | NA |
| 21/04/09 | NA | NA | NA | NA | NA |
| 28/04/09 | NA | NA | NA | NA | NA |
| 05/05/09 | 37.34 | 16.71 | 2231.7 | 2534 | 3.25 |
| 12/05/09 | 37.09 | 17.63 | 2235.8 | 2540.6 | 3.30 |
| 19/05/09 | 37.42 | 19.89 | 2228 | 2535.3 | 3.35 |
| 26/05/09 | 36.84 | 22.09 | 2232.2 | 2541 | 3.44 |
| 02/06/09 | 37.54 | 20.86 | 2230.7 | 2532.4 | 3.31 |
| 09/06/09 | 37.6 | 18.27 | 2241.7 | 2535 | 3.18 |
| 16/06/09 | 37.63 | 22.84 | 2216.2 | 2521.5 | 3.37 |
| 23/06/09 | 37.75 | 22.76 | 2221.5 | 2534.5 | 3.45 |
| 30/06/09 | 37.81 | 23.26 | 2225.3 | 2541 | 3.49 |
| 07/07/09 | 37.64 | 24.90666667 | 2224.5 | 2542 | 3.55 |
| 13/07/09 | 37.82 | 24.15 | 2218.8 | 2533.9 | 3.50 |
| 21/07/09 | 37.8 | 22.71 | 2214.6 | 2532.6 | 3.50 |
| 28/07/09 | NA | NA | NA | NA | NA |
| 04/08/09 | 38 | 23.79 | 2216.4 | 2539.8 | 3.57 |
| 11/08/09 | 37.99 | 24.6 | 2226 | 2544.5 | 3.54 |
| 18/08/09 | NA | NA | NA | NA | NA |
| 25/08/09 | 38.03 | 26.84 | 2228.6 | 2554.1 | 3.66 |
| 01/09/09 | 38.11 | 26.84 | 2224.5 | 2555 | 3.71 |
| 08/09/09 | 38.2 | 24.22 | NA | 2540.8 | NA |
| 15/09/09 | NA | NA | NA | NA | NA |
| 22/09/09 | NA | NA | NA | NA | NA |
| 29/09/09 | NA | NA | NA | NA | NA |
| 06/10/09 | NA | NA | NA | NA | NA |
| 13/10/09 | NA | NA | NA | NA | NA |
| 20/10/09 | NA | NA | NA | NA | NA |
| 29/10/09 | 38.1 | 21.15 | 2211.6 | 2553.7 | 3.70 |
| 05/11/09 | 37.985 | 18.97 | 2214.3 | 2548.5 | 3.58 |
| 10/11/09 | 38.02 | 18.66 | 2217.7 | 2549.8 | 3.55 |
| 17/11/09 | 37.98 | 18.11 | 2208.8 | 2542.2 | 3.56 |
| 24/11/09 | 37.99 | 17.59 | 2219.9 | 2539.3 | 3.41 |
| 01/12/09 | 37.9 | 17.05 | 2224.1 | 2538.6 | 3.36 |
| 08/12/09 | 37.92 | 16.74 | 2227.1 | 2546 | 3.40 |
| 15/12/09 | 37.97 | 15.99 | 2229 | 2547.6 | 3.38 |
| 22/12/09 | 37.86 | 14.51 | 2230.3 | 2538.2 | 3.26 |
| 29/12/09 | NA | NA | NA | NA | NA |
| 05/01/10 | 37.85 | 14.21 | 2240.3 | 2543.9 | 3.21 |
| 12/01/10 | 37.88 | 14 | 2246.5 | 2541.8 | 3.13 |
| 19/01/10 | 37.87 | 13.63 | 2250.3 | 2547 | 3.14 |
| 25/01/10 | 37.72 | 13.07 | 2246.6 | 2532.2 | 3.02 |
| 02/02/10 | 37.94 | 13.22 | 2255.2 | 2546.2 | 3.08 |
| 11/02/10 | 37.92 | 13.2 | 2251.4 | 2543.9 | 3.09 |
| 16/02/10 | 37.96 | 13.2 | 2254.4 | 2546.5 | 3.09 |
| 23/02/10 | NA | NA | NA | NA | NA |
| 02/03/10 | NA | NA | NA | NA | NA |
| 09/03/10 | 37.5 | 12.51 | 2249.7 | 2542.1 | 3.09 |
| 16/03/10 | 37.86 | 13 | 2254 | 2545.4 | 3.08 |
| 23/03/10 | 37.86 | 13.27 | 2253.7 | 2542.2 | 3.05 |
| 30/03/10 | 37.84 | 13.82 | 2246.8 | 2542 | 3.13 |
| 06/04/10 | 37.72 | 13.94 | 2245.7 | 2544.3 | 3.17 |
| 13/04/10 | 37.74 | 13.86 | 2252.2 | 2538.4 | 3.04 |
| 20/04/10 | 37.79 | 14.78 | 2240.3 | 2541.4 | 3.20 |
| 27/04/10 | 37.78 | 15.16 | 2232.1 | 2540.5 | 3.27 |
| 04/05/10 | NA | NA | NA | NA | NA |
| 11/05/10 | 37.7 | 16.39 | 2234.7 | 2539.8 | 3.26 |
| 18/05/10 | 37.63 | 16.66 | 2218.4 | 2538.9 | 3.42 |
| 25/05/10 | 37.49 | 18.06 | 2230.4 | 2545 | 3.39 |
| 01/06/10 | NA | NA | NA | NA | NA |
| 08/06/10 | 37.46 | 21.47 | 2216 | 2542.9 | 3.58 |
| 15/06/10 | 37.65 | 21.38 | 2219 | 2539.9 | 3.51 |
| 23/06/10 | 37.52 | 21.83 | 2218.3 | 2540.5 | 3.54 |
| 29/06/10 | 37.26 | 23.56 | 2216.5 | 2539 | 3.58 |
| 06/07/10 | 37.62 | 24.75 | 2217.2 | 2545 | 3.65 |
| 13/07/10 | 37.47 | 24.62 | 2213 | 2545.9 | 3.71 |
| 20/07/10 | 38 | 25 | 2220.1 | 2558.8 | 3.76 |
| 27/07/10 | 38.01 | 25.33 | 2219.7 | 2559.1 | 3.77 |
| 03/08/10 | 37.98 | 25.35 | 2226 | 2563.7 | 3.76 |
| 10/08/10 | NA | NA | NA | NA | NA |
| 17/08/10 | 37.91 | 21.21 | 2224.1 | 2552.3 | 3.57 |
| 24/08/10 | 37.97 | 24.58 | 2212.9 | 2554.6 | 3.78 |
| 31/08/10 | 38.06 | 23.92 | 2224.3 | 2558.3 | 3.68 |
| 07/09/10 | NA | NA | NA | NA | NA |
| 14/09/10 | 38.02 | 22.97 | 2216.3 | 2557.2 | 3.73 |
| 21/09/10 | 38.02 | 22.33 | 2226.3 | 2558.9 | 3.64 |
| 28/09/10 | 38.15 | 22.24 | 2220.6 | 2564.7 | 3.74 |
| 07/10/10 | 38.11 | 20.84 | 2222.6 | 2563.5 | 3.69 |
| 12/10/10 | 38.14 | 19.79 | 2229.6 | 2566.9 | 3.63 |
| 19/10/10 | NA | NA | NA | NA | NA |
| 26/10/10 | NA | NA | NA | NA | NA |
| 03/11/10 | 36.9 | 18.05 | 2225.2 | 2549.1 | 3.51 |
| 09/11/10 | 38.03 | 18.32 | 2240.5 | 2567.5 | 3.51 |
| 17/11/10 | 38 | 17.82 | 2234.8 | 2561.3 | 3.49 |
| 23/11/10 | 37.62 | 17.08 | 2242.9 | 2560.3 | 3.41 |
| 30/11/10 | 38 | 15 | 2244.8 | 2559.3 | 3.33 |
| 08/12/10 | 38 | 14.5 | 2242.2 | 2554.6 | 3.30 |
| 14/12/10 | 38 | 14 | 2239.2 | 2563.5 | 3.41 |
| 21/12/10 | 37.81 | 14.67 | 2245.9 | 2559.6 | 3.33 |
| 28/12/10 | 37.43 | 13.88 | 2248.7 | 2551.1 | 3.22 |
| 04/01/11 | 37.75 | 14.09 | 2242.8 | 2544.5 | 3.20 |
| 11/01/11 | 37.62 | 13.6 | 2249 | 2548.3 | 3.17 |
| 18/01/11 | 37.46 | 13.3 | 2247.1 | 2550.4 | 3.21 |
| 25/01/11 | 37.85 | 13.34 | 2259.8 | 2550.3 | 3.08 |
| 01/02/11 | 37.99 | 13.425 | 2262.9 | 2554.6 | 3.09 |
| 08/02/11 | 37.96 | 13.26 | 2256.7 | 2554.1 | 3.14 |
| 15/02/11 | 37.7 | 13.38 | 2239.7 | 2533.2 | 3.11 |
| 22/02/11 | 37.88 | 13.1 | 2255.1 | 2546.4 | 3.08 |
| 01/03/11 | 38.05 | 13.38 | 2259.6 | 2554.3 | 3.11 |
| 08/03/11 | NA | NA | NA | NA | NA |
| 15/03/11 | 38.02 | 13.12 | 2257.4 | 2553.3 | 3.12 |
| 22/03/11 | 37.23 | 13.72 | 2258.7 | 2551.9 | 3.13 |
| 29/03/11 | 37.41 | 13.65 | 2239.2 | 2544.8 | 3.24 |
| 05/04/11 | 37.3 | 14.99 | 2251 | 2552.8 | 3.23 |
| 12/04/11 | NA | NA | NA | NA | NA |
| 19/04/11 | 37.64 | 15.72 | 2240 | 2555.8 | 3.37 |
| 26/04/11 | 37.79 | 15.57 | 2247.2 | 2555.6 | 3.29 |
| 03/05/11 | 37.78 | 16.64 | 2248.1 | 2557.9 | 3.32 |
| 10/05/11 | 37.81 | 17.83 | 2244.9 | 2559.6 | 3.39 |
| 17/05/11 | 37.72 | 18.31 | 2253 | 2561 | 3.33 |
| 24/05/11 | 37.89 | 19.53 | 2238.9 | 2557.2 | 3.45 |
| 31/05/11 | 37.92 | 19.93 | 2237 | 2555.2 | 3.45 |
| 07/06/11 | 37.78 | 20.38 | 2235 | 2543.4 | 3.36 |
| 14/06/11 | 37.95 | 21.2 | 2232.3 | 2548.7 | 3.45 |
| 21/06/11 | 37.89 | 21.51 | 2234.8 | 2549.1 | 3.44 |
| 28/06/11 | NA | NA | NA | NA | NA |
| 05/07/11 | 37.84 | 24.5 | 2227 | 2544.3 | 3.53 |
| 12/07/11 | 37.98 | 25.27 | 2221.4 | 2545.5 | 3.61 |
| 19/07/11 | 38.01 | 20.26 | 2227.2 | 2546.2 | 3.46 |
| 26/07/11 | 38.03 | 23.58 | 2217.5 | 2547.9 | 3.64 |
| 02/08/11 | 37.99 | 23.82 | 2222.5 | 2545.6 | 3.57 |
| 09/08/11 | 38.03 | 21.72 | 2223.1 | 2543.7 | 3.50 |
| 16/08/11 | 38.11 | 24.18 | 2202.3 | 2552.3 | 3.84 |
| 23/08/11 | 38.11 | 25.91 | 2236.7 | 2541.9 | 3.43 |
| 30/08/11 | 38.19 | 24.46 | 2230.5 | 2557.5 | 3.62 |
| 06/09/11 | 38.25 | 24.48 | 2227.4 | 2554.5 | 3.61 |
| 13/09/11 | 38.25 | 25.15 | 2223.9 | 2556 | 3.68 |
| 20/09/11 | NA | NA | NA | NA | NA |
| 27/09/11 | 38.21 | 22.81 | 2222.9 | 2555.1 | 3.63 |
| 04/10/11 | 38.23 | 22.9 | 2226.3 | 2558.5 | 3.63 |
| 11/10/11 | 38.2 | 21.2 | 2292.1 | 2561.8 | 3.01 |
| 18/10/11 | 38.18 | 20 | 2225.8 | 2560.2 | 3.60 |
| 26/10/11 | 38.18 | 19.12 | 2227.5 | 2549.4 | 3.46 |
| 02/11/11 | 38.23 | 19.39 | 2229.4 | 2555.8 | 3.51 |
| 10/11/11 | 37.85 | 18.45 | 2230 | 2552.2 | 3.46 |
| 15/11/11 | NA | NA | NA | NA | NA |
| 22/11/11 | 38.13 | 18.12 | 2227.4 | 2553.2 | 3.48 |
| 29/11/11 | 38.08 | 17.81 | 2229.3 | 2554.6 | 3.47 |
| 06/12/11 | 38.09 | 17.55 | 2231.8 | 2553.9 | 3.44 |
| 13/12/11 | 38.1 | 17.28 | 2234.7 | 2554.9 | 3.42 |
| 19/12/11 | 38.08 | 15.34 | 2246.6 | 2554.7 | 3.27 |
| 27/12/11 | NA | NA | NA | NA | NA |
| 03/01/12 | 38.09 | 15.07 | 2244.9 | 2553.7 | 3.27 |
| 10/01/12 | 38.09 | 15.11 | 2266 | 2560.4 | 3.14 |
| 17/01/12 | 38.1 | 14.8 | 2247.4 | 2553.2 | 3.24 |
| 24/01/12 | NA | NA | NA | NA | NA |
| 31/01/12 | 38.09 | 14.37 | 2251.5 | 2552.3 | 3.18 |
| 07/02/12 | NA | NA | NA | NA | NA |
| 14/02/12 | 38.21 | 13.15 | 2280.8 | 2565.2 | 3.01 |
| 21/02/12 | 38.15 | 13.4 | 2266.3 | 2560 | 3.10 |
| 28/02/12 | 38.17 | 13.48 | 2266.8 | 2562.1 | 3.12 |
| 06/03/12 | 38.12 | 13.39 | 2260.8 | 2558.1 | 3.14 |
| 13/03/12 | 38.14 | 13.47 | 2265 | 2560.6 | 3.12 |
| 20/03/12 | 38.15 | 13.47 | 2263.7 | 2563.2 | 3.16 |
| 27/03/12 | 38.14 | 14.28 | 2262.3 | 2560.9 | 3.16 |
| 03/04/12 | 38.13 | 15.24 | 2256 | 2561.8 | 3.25 |
| 10/04/12 | 38.16 | 14.55 | 2265 | 2560.4 | 3.14 |
| 17/04/12 | 38.03 | 14.79 | 2252.8 | 2567.3 | 3.33 |
| 25/04/12 | 38.09 | 13.79 | 2273.9 | 2566.9 | 3.11 |
| 02/05/12 | NA | NA | NA | NA | NA |
| 09/05/12 | 37.63 | 16.74 | 2256.9 | 2555.9 | 3.22 |
| 15/05/12 | 38.02 | 17.45 | 2256.5 | 2562.2 | 3.29 |
| 22/05/12 | 37.99 | 16.7 | 2257.4 | 2561.3 | 3.26 |
| 29/05/12 | 37.94 | 18.98 | 2255.3 | 2561.9 | 3.32 |
| 05/06/12 | 37.76 | 20.66 | 2250.1 | 2551.9 | 3.31 |
| 13/06/12 | 38.12 | 16.19 | 2251.4 | 2559.6 | 3.29 |
| 19/06/12 | 37.97 | 23.22 | 2244 | 2563.8 | 3.53 |
| 26/06/12 | 37.99 | 23.84 | 2236.2 | 2560.9 | 3.59 |
| 03/07/12 | 37.97 | 25.58 | 2251.3 | 2571.7 | 3.59 |
| 10/07/12 | 38.16 | 26.11 | 2238.1 | 2567.6 | 3.69 |
| 17/07/12 | 38.12 | 21.93 | 2249.8 | 2565 | 3.46 |
| 24/07/12 | 38.21 | 23.03 | 2240 | 2565 | 3.57 |
| 31/07/12 | 38.23 | 23.3 | 2246.4 | 2569.8 | 3.56 |
| 07/08/12 | 38.31 | 25.27 | 2261.7 | 2568.6 | 3.44 |
| 14/08/12 | NA | NA | NA | NA | NA |
| 21/08/12 | 38.37 | 26.74 | 2249.3 | 2576 | 3.67 |
| 28/08/12 | 38.35 | 25.88 | 2242.7 | 2580 | 3.76 |
| 05/09/12 | 38.39 | 24.26 | 2247.1 | 2577.2 | 3.65 |
| 11/09/12 | 38.35 | 24.02 | 2250.6 | 2566.2 | 3.49 |
| 18/09/12 | 38.38 | 23.04 | 2239.1 | 2576.1 | 3.69 |
| 25/09/12 | 38.35 | 22.31 | 2239.4 | 2574 | 3.65 |
| 02/10/12 | 38.23 | 21.85 | 2231.1 | 2565.6 | 3.64 |
| 09/10/12 | 38.32 | 22.02 | 2231.1 | 2572.3 | 3.71 |
| 16/10/12 | 38.22 | 21.47 | 2224.4 | 2561.7 | 3.72 |
| 23/10/12 | 38.28 | 20.78 | 2235.1 | 2570.1 | 3.62 |
| 30/10/12 | 38.24 | 20.02 | 2242.9 | 2572.5 | 3.56 |
| 06/11/12 | 38.1 | 19 | 2240.8 | 2557.3 | 3.41 |
| 13/11/12 | 38.02 | 18.05 | 2234 | 2546.9 | 3.45 |
| 20/11/12 | 38.08 | 17.46 | 2243.4 | 2564.9 | 3.44 |
| 27/11/12 | 38.12 | 17.47 | 2245.7 | 2564.7 | 4.24 |
| 05/12/12 | 38.11 | 16.92 | 2237.5 | 2559.3 | 3.43 |
| 11/12/12 | 38.12 | 16.32 | 2228.4 | 2544.7 | 3.36 |
| 18/12/12 | 38.09 | 15.81 | 2246.4 | 2558.5 | 3.32 |
| 28/12/12 | 38 | 15 | 2255 | 2560.8 | 3.29 |

**Supplementary Figures**
