# Supplementary material for: Comparison of Mediterranean Pteropod Shell Biometrics and Ultrastructure from Historical (1910 and 1921) and Present Day (2012) Samples Provides Baseline for Monitoring Effects of Global Change
Source: PLoS One. 2017 Jan 26;12(1):e0167891. doi: 10.1371/journal.pone.0167891 (PMC5268398; doi:10.1371/journal.pone.0167891)
Supplement: S2 Table — (DOCX) [file pone.0167891.s007.docx]

S2 Table: Modern specimens used in analysis of shell morphometrics based on ontogenetic stage.

| **Species** | **Average thickness (µm)** | **Thickness standard deviation**  **(µm)** | **Shell surface area (mm^2^)** | **Weight (mg)** | **Volume of shell CaCO_3_**  **(mm^3^)** | **Shell density**  **(mg mm^-3^)** | **Shell length**  **(mm)** | **Shell width (mm)** |
| --- | --- | --- | --- | --- | --- | --- | --- | --- |
| *S. subula* | 13.27 | 3.62 | 27.81 | NA | 0.37 | NA | 1.97 | 0.56 |
| *S. subula* | 14.72 | 4.25 | 24.15 | NA | 0.36 | NA | 2.10 | 0.56 |
| *S. subula* | 14.96 | 3.98 | 22.11 | 0.11 | 0.33 | 0.34 | 3.12 | 0.77 |
| *S. subula* | 51.19 | 20.77 | 56.3 | 0.59 | 2.88 | 0.21 | 3.13 | 0.78 |
| *S. subula* | 16.02 | 5.1 | 73.17 | 0.50 | 1.17 | 0.42 | 3.42 | 0.84 |
| *S. subula* | 16.44 | 3.69 | 12.41 | 0.14 | 0.20 | 0.71 | 3.45 | 0.90 |
| *S. subula* | 18.06 | 4.72 | 21.76 | 0.16 | 0.39 | 0.41 | 3.57 | 0.85 |
| *S. subula* | 14.64 | 3.84 | 13.16 | 0.28 | 0.19 | 1.47 | 3.78 | 0.91 |
| *S. subula* | 24.47 | 8.03 | NA | 0.24 | NA | NA | 3.80 | 0.93 |
| *S. subula* | 19.25 | 5.2 | 59.03 | 0.36 | 1.14 | 0.31 | 4.10 | 1.01 |
| *S. subula* | 15.95 | 4.78 | 16.41 | 0.17 | 0.26 | 0.65 | 4.13 | 0.99 |
| *S. subula* | 19.57 | 5.6 | 51.77 | 0.42 | 1.01 | 0.42 | 4.36 | 1.01 |
| *S. subula* | 17.81 | 4.69 | 23.14 | 0.63 | 0.41 | 1.52 | 4.37 | 1.05 |
| *S. subula* | 21.52 | 4.91 | NA | 0.29 | NA | NA | 4.41 | 1.11 |
| *S. subula* | 19.53 | 5.71 | 42.14 | NA | 0.82 | NA | 4.42 | 1.05 |
| *S. subula* | 22.4 | 7.3 | 90.91 | 0.42 | 2.04 | 0.21 | 4.59 | 1.10 |
| *S. subula* | 20.15 | 5.11 | 54.57 | 0.45 | 1.10 | 0.40 | 4.81 | 1.16 |
| *S. subula* | 46.58 | 14.02 | 51.27 | 0.29 | 2.39 | 0.12 | 4.87 | 1.16 |
| *S. subula* | 21.61 | 6.78 | 81.29 | NA | 1.76 | NA | 4.90 | 1.12 |
| *S. subula* | 37.54 | 12.12 | 30.85 | 0.43 | 1.16 | 0.37 | 4.91 | 1.23 |
| *S. subula* | 23.9 | 6.01 | 54.34 | 0.42 | 1.30 | 0.33 | 4.92 | 1.16 |
| *S. subula* | 20.15 | 8.83 | NA | 0.53 | NA | NA | 5.12 | 1.18 |
| *S. subula* | 41.3 | 14.1 | 94.66 | 0.73 | 3.91 | 0.19 | 5.23 | 1.27 |
| *S. subula* | 22.38 | 5.38 | NA | NA | NA | NA | 5.25 | 1.24 |
| *S. subula* | 20.77 | 5.05 | 28.08 | 0.55 | 0.58 | 0.95 | 5.33 | 1.19 |
| *S. subula* | 39.22 | 8.73 | 33.1 | 0.45 | 1.30 | 0.35 | 5.42 | 1.30 |
| *S. subula* | 31.61 | 7.55 | 25.4 | 0.61 | 0.80 | 0.76 | 5.50 | 1.30 |
| *S. subula* | 52.67 | 19.54 | 34.51 | 0.50 | 1.82 | 0.27 | 5.51 | 1.35 |
| *S. subula* | 45.05 | 8.83 | 28.05 | 0.50 | 1.26 | 0.39 | 5.58 | 1.30 |
| *S. subula* | 29.54 | 6.91 | 38.1 | 0.67 | 1.13 | 0.59 | 5.58 | 1.32 |
| *S. subula* | 47.63 | 17.17 | 45.05 | 0.45 | 2.15 | 0.21 | 5.59 | 1.35 |
| *S. subula* | 54.54 | 22.35 | 48.61 | 0.55 | 2.65 | 0.21 | 5.63 | 1.32 |
| *S. subula* | NA | NA | NA | 0.60 | NA | NA | NA | 1.39 |
| *S. subula* | 44.92 | 13.67 | 41.68 | 0.55 | 1.87 | 0.30 | 5.85 | 1.38 |
| *S. subula* | 58.1 | 20.59 | 52.02 | 0.62 | 3.02 | 0.21 | 5.87 | 1.32 |
| *S. subula* | 21.73 | 5.27 | 41.57 | 0.34 | 0.90 | 0.38 | 5.91 | 1.34 |
| *S. subula* | 21.36 | 5.22 | NA | 0.44 | NA | NA | 5.92 | 1.30 |
| *S. subula* | 52.43 | 20.56 | 90.55 | 0.67 | 4.75 | 0.14 | 5.94 | 1.45 |
| *S. subula* | 48.94 | 15.76 | 46.47 | 0.69 | 2.27 | 0.30 | 5.96 | 1.37 |
| *S. subula* | 44.56 | 16.02 | 47.31 | 0.66 | 2.11 | 0.31 | 5.98 | 1.36 |
| *S. subula* | 23.08 | 6.26 | 86.88 | 0.65 | 2.01 | 0.32 | 6.07 | 1.33 |
| *S. subula* | 42.25 | 14.15 | 77.69 | 0.51 | 3.28 | 0.15 | 6.09 | 1.43 |
| *S. subula* | 27.88 | 5.88 | 28.81 | 0.44 | 0.80 | 0.55 | 6.14 | 1.41 |
| *S. subula* | 22.61 | 5.27 | 48.92 | 0.43 | 1.11 | 0.39 | 6.63 | 1.44 |
| *S. subula* | 21.26 | 5.37 | 44.98 | 0.20 | 0.96 | 0.21 | 6.68 | 1.49 |
| *S. subula* | 39.76 | 11.47 | 60.19 | 1.03 | 2.39 | 0.43 | 6.72 | 1.53 |
| *S. subula* | 37.49 | 7.92 | 51.59 | 0.70 | 1.93 | 0.36 | 6.85 | 1.51 |
| *S. subula* | 25.61 | 5.9 | 40.38 | 0.93 | 1.03 | 0.90 | 7.04 | 1.56 |
| *S. subula* | 34.96 | 10.42 | 55.62 | 1.05 | 1.94 | 0.54 | 7.10 | 1.64 |
| *S. subula* | 36.65 | 10.34 | 58.22 | 0.67 | 2.13 | 0.31 | 7.23 | 1.55 |
| *S. subula* | 41.14 | 11.38 | 68.95 | 0.86 | 2.84 | 0.30 | 7.33 | 1.82 |
| *S. subula* | 42.72 | 19.07 | 62.18 | 0.59 | 2.66 | 0.22 | 7.44 | 1.75 |
| *S. subula* | 41.84 | 12.25 | 57.85 | 1.16 | 2.42 | 0.48 | 7.56 | 1.53 |
| *S. subula* | 49.96 | 18.33 | 88.36 | 0.86 | 4.41 | 0.20 | 7.69 | 1.59 |
| *S. subula* | 17.31 | 4.98 | NA | 0.52 | NA | NA | NA | NA |
| *S. subula* | 15.24 | 4.23 | NA | 0.62 | NA | NA | NA | NA |
| *S. subula* | 21.22 | 6.34 | 58.98 | 0.57 | 1.25 | 0.46 | NA | NA |
| *S. subula* | 15.95 | 6.04 | 12.13 | NA | 0.19 | NA | NA | NA |
| *C. inflexa* | 22.08 | 5.9 | NA | 1.56 | NA | NA | NA | 4.82 |
| *C. inflexa* | NA | 5.26 | NA | 1.75 | NA | NA | NA | NA |
| *C. inflexa* | 25.87 | 6.75 | NA | 2.09 | NA | NA | NA | 5.45 |
| *C. inflexa* | 28.93 | 8.46 | 62.94 | 2.16 | 1.82 | 1.18 | 5.63 | 3.31 |
| *C. inflexa* | 23.19 | 5.19 | 62.62 | 1.42 | 1.45 | 0.98 | 5.53 | 3.23 |
| *C. inflexa* | 30.51 | 7.99 | 64.27 | 2.14 | 1.96 | 1.09 | 5.46 | 3.93 |
| *C. inflexa* | 35.46 | 11.54 | 61.97 | 2.55 | 2.20 | 1.16 | 5.92 | 3.53 |
| *C. inflexa* | 27.25 | 7.77 | 59.98 | 1.84 | 1.63 | 1.12 | 5.59 | 3.69 |
| *C. inflexa* | 22.34 | 4.91 | 62.68 | 1.57 | 1.40 | 1.12 | 5.66 | 3.61 |
| *C. inflexa* | 30.67 | 10.94 | 68.95 | 2.11 | 2.11 | 1.00 | 5.98 | 3.22 |
| *C. inflexa* | 30.6 | 7.96 | 67.99 | 2.54 | 2.08 | 1.22 | 5.80 | 3.73 |
| *C. inflexa* | 20.53 | 4.51 | 59.79 | 1.02 | 1.23 | 0.83 | 5.60 | 3.36 |
| *C. inflexa* | 19.06 | 4.55 | 61.73 | NA | 1.18 | NA | 5.73 | 3.89 |
| *C. inflexa* | 27.36 | 6.43 | 57.79 | 1.84 | 1.58 | 1.16 | 5.68 | 3.30 |
| *C. inflexa* | 26.06 | 2.61 | 54.48 | 1.45 | 1.42 | 1.02 | 5.12 | 3.12 |
| *C. inflexa* | 24.65 | 6.41 | 58.43 | 1.72 | 1.44 | 1.20 | 5.53 | 3.26 |
| *C. inflexa* | 20.43 | 4.34 | 55.04 | 1.12 | 1.12 | 1.00 | 5.52 | 3.41 |
| *C. inflexa* | 27.22 | 7.65 | 55.75 | 1.69 | 1.52 | 1.12 | 5.34 | NA |
| *C. inflexa* | 19.39 | 4.13 | 58.77 | 1.29 | 1.14 | 1.13 | 5.61 | 3.41 |
| *C. inflexa* | 17.68 | 7.23 | 65.53 | NA | 1.16 | NA | 5.17 | NA |
| *C. inflexa* | 19.35 | 4.86 | 54.27 | NA | 1.05 | NA | 5.27 | 3.89 |
| *C. inflexa* | 17.57 | 3.67 | 50.81 | NA | 0.89 | NA | NA | NA |
| *C. inflexa* | 37.37 | 13.29 | 59.58 | 2.63 | 2.23 | 1.18 | 5.48 | 3.56 |
| *C. inflexa* | 29.91 | 8.61 | 59.27 | 2.00 | 1.77 | 1.13 | 5.32 | 3.20 |
| *C. inflexa* | 32.17 | 10.84 | 53.36 | 2.05 | 1.72 | 1.20 | 5.73 | 3.05 |
| *C. inflexa* | 21.23 | 6.39 | 67.63 | 1.98 | 1.44 | 1.38 | 5.78 | 3.39 |
| *C. inflexa* | 30.08 | 11.52 | 47.82 | 1.71 | 1.44 | 1.19 | 5.06 | 3.05 |
| *C. inflexa* | 21.96 | 6.38 | 56.88 | 1.30 | 1.25 | 1.04 | 5.26 | 3.75 |
| *C. inflexa* | 30.72 | 10.18 | 47.51 | 1.72 | 1.46 | 1.18 | 5.15 | 2.91 |
| *C. inflexa* | 15.2 | 4.26 | 32.81 | 0.96 | 0.50 | 1.93 | 3.46 | 3.07 |
| *C. inflexa* | 18.67 | 4.48 | 47.1 | NA | 0.88 | NA | 4.65 | 3.88 |
| *C. inflexa* | 13.04 | 4.42 | 16.35 | 0.07 | 0.21 | 0.33 | 2.78 | 2.20 |
| *C. inflexa* | NA | NA | NA | NA | NA | NA | NA | NA |
| *C. inflexa* | 15.75 | 4.55 | 37.76 | 0.83 | 0.59 | 1.40 | 4.28 | 3.45 |
| *C. inflexa* | 17.87 | 3.49 | 43.05 | NA | 0.77 | NA | 4.59 | 3.74 |
| *C. inflexa* | 11.62 | 3.65 | 8.32 | 0.21 | 0.10 | 2.15 | 2.68 | 1.61 |
| *C. inflexa* | 14.79 | 4.2 | 24.66 | NA | 0.36 | NA | 3.14 | 2.61 |
| *C. inflexa* | 36.29 | 13.48 | 52.49 | 2.66 | 1.90 | 1.40 | 5.58 | 4.62 |
| *C. inflexa* | 26.45 | 7.36 | 53.28 | 1.71 | 1.41 | 1.21 | 5.25 | 4.20 |
| *C. inflexa* | 13.09 | 4.23 | 22.63 | NA | 0.30 | NA | 2.95 | 2.48 |
| *C. inflexa* | 11.52 | 3.84 | 20.95 | 0.24 | 0.24 | 1.00 | 2.93 | 2.53 |
| *C. inflexa* | 17.59 | 4.59 | 35.98 | 0.16 | 0.63 | 0.25 | 3.83 | 3.19 |
| *C. inflexa* | 14.87 | 4.56 | NA | NA | NA | NA | 2.31 | NA |
| *C. inflexa* | 18.37 | 4.39 | 25.72 | NA | NA | NA | 3.10 | 2.50 |
| *C. inflexa* | 20.76 | 5.11 | 41.09 | NA | 0.85 | NA | 3.94 | 3.24 |
| *C. inflexa* | 20.96 | 4.35 | 47.3 | 0.45 | 0.99 | 0.46 | NA | 3.63 |
| *C. inflexa* | 18.85 | 4.83 | 39.18 | NA | 0.74 | NA | 4.09 | 3.32 |
| *C. inflexa* | 20.93 | 5.25 | 37.4 | NA | 0.78 | NA | 3.26 | 3.09 |
| *C. inflexa* | 19.53 | 5.76 | 27.53 | NA | 0.54 | NA | 3.18 | NA |
| *C. inflexa* | 19.37 | 5.22 | 33.12 | NA | 0.64 | NA | 3.16 | 2.27 |
| *C. inflexa* | 16.76 | 4.05 | 23.98 | NA | 0.40 | NA | NA | NA |
| *C. inflexa* | 19.04 | 4.48 | 18.08 | NA | 0.34 | NA | 3.91 | NA |
| *C. inflexa* | 47.69 | 21.61 | 81.13 | 3.93 | 3.87 | 1.02 | 5.85 | 4.73 |
| *C. inflexa* | 19.31 | 6.08 | 65.71 | 2.82 | 1.27 | 2.22 | 4.89 | 4.05 |
| *C. inflexa* | 34.35 | 11.03 | 66.7 | 1.18 | 2.29 | 0.51 | 5.94 | 3.19 |
| *C. inflexa* | 26.62 | 4.87 | 53.08 | 1.08 | 1.41 | 0.76 | 4.08 | 3.76 |
| *C. inflexa* | 17.03 | 4.71 | 25.67 | 0.17 | 0.44 | 0.38 | 2.95 | 2.62 |
| *C. inflexa* | 40.06 | 12.61 | 71.86 | NA | 2.88 | NA | 5.77 | NA |
| *C. inflexa* | 23.49 | 6.11 | 61.37 | NA | 1.44 | NA | NA | NA |
