# Supplementary material for: Comparison of Mediterranean Pteropod Shell Biometrics and Ultrastructure from Historical (1910 and 1921) and Present Day (2012) Samples Provides Baseline for Monitoring Effects of Global Change
Source: PLoS One. 2017 Jan 26;12(1):e0167891. doi: 10.1371/journal.pone.0167891 (PMC5268398; doi:10.1371/journal.pone.0167891)
Supplement: S3 Table — A size filter has been applied to the modern samples to select those within the same length and width range as the museum samples. “New” denotes samples from 2012 and “Old” denotes museum samples. (DOCX) [file pone.0167891.s008.docx]

S3 Table: The modern and museum specimens used for comparison. A size filter has been applied to the modern samples to select those within the same length and width range as the museum samples. “New” denotes samples from 2012 and “Old” denotes museum samples.

| **Sample** | **Species** | **Average thickness (µm)** | **Thickness standard deviation**  **(µm)** | **Shell surface area (mm^2^)** | **Weight (mg)** | **Volume of shell CaCO_3_**  **(mm^3^)** | **Shell density**  **(mg mm^-3^)** | **Shell length**  **(mm)** | **Shell width (mm)** |
| --- | --- | --- | --- | --- | --- | --- | --- | --- | --- |
| New | *S. subula* | 17.81 | 4.69 | 23.14 | 0.63 | 0.41 | 1.52 | 4.37 | 1.05 |
| New | *S. subula* | 21.52 | 4.91 | NA | 0.29 | NA | NA | 4.41 | 1.11 |
| New | *S. subula* | 19.53 | 5.71 | 42.14 | NA | 0.82 | NA | 4.42 | 1.05 |
| New | *S. subula* | 22.4 | 7.3 | 90.91 | 0.42 | 2.04 | 0.21 | 4.59 | 1.10 |
| New | *S. subula* | 20.15 | 5.11 | 54.57 | 0.45 | 1.10 | 0.40 | 4.81 | 1.16 |
| New | *S. subula* | 46.58 | 14.02 | 51.27 | 0.29 | 2.39 | 0.12 | 4.87 | 1.16 |
| New | *S. subula* | 21.61 | 6.78 | 81.29 | NA | 1.76 | NA | 4.90 | 1.12 |
| New | *S. subula* | 37.54 | 12.12 | 30.85 | 0.43 | 1.16 | 0.37 | 4.91 | 1.23 |
| New | *S. subula* | 23.9 | 6.01 | 54.34 | 0.42 | 1.30 | 0.33 | 4.92 | 1.16 |
| New | *S. subula* | 20.15 | 8.83 | NA | 0.53 | NA | NA | 5.12 | 1.18 |
| New | *S. subula* | 41.3 | 14.1 | 94.66 | 0.73 | 3.91 | 0.19 | 5.23 | 1.27 |
| New | *S. subula* | 22.38 | 5.38 | NA | NA | NA | NA | 5.25 | 1.24 |
| New | *S. subula* | 20.77 | 5.05 | 28.08 | 0.55 | 0.58 | 0.95 | 5.33 | 1.19 |
| New | *S. subula* | 39.22 | 8.73 | 33.1 | 0.45 | 1.30 | 0.35 | 5.42 | 1.30 |
| New | *S. subula* | 31.61 | 7.55 | 25.4 | 0.61 | 0.80 | 0.76 | 5.50 | 1.30 |
| New | *S. subula* | 52.67 | 19.54 | 34.51 | 0.50 | 1.82 | 0.27 | 5.51 | 1.35 |
| New | *S. subula* | 45.05 | 8.83 | 28.05 | 0.50 | 1.26 | 0.39 | 5.58 | 1.30 |
| New | *S. subula* | 29.54 | 6.91 | 38.1 | 0.67 | 1.13 | 0.59 | 5.58 | 1.32 |
| New | *S. subula* | 47.63 | 17.17 | 45.05 | 0.45 | 2.15 | 0.21 | 5.59 | 1.35 |
| New | *S. subula* | 54.54 | 22.35 | 48.61 | 0.55 | 2.65 | 0.21 | 5.63 | 1.32 |
| New | *S. subula* | 44.92 | 13.67 | 41.68 | 0.55 | 1.87 | 0.30 | 5.85 | 1.38 |
| New | *S. subula* | 58.1 | 20.59 | 52.02 | 0.62 | 3.02 | 0.21 | 5.87 | 1.32 |
| New | *S. subula* | 21.73 | 5.27 | 41.57 | 0.34 | 0.90 | 0.38 | 5.91 | 1.34 |
| New | *S. subula* | 21.36 | 5.22 | NA | 0.44 | NA | NA | 5.92 | 1.30 |
| New | *S. subula* | 52.43 | 20.56 | 90.55 | 0.67 | 4.75 | 0.14 | 5.94 | 1.45 |
| New | *S. subula* | 48.94 | 15.76 | 46.47 | 0.69 | 2.27 | 0.30 | 5.96 | 1.37 |
| New | *S. subula* | 44.56 | 16.02 | 47.31 | 0.66 | 2.11 | 0.31 | 5.98 | 1.36 |
| New | *S. subula* | 23.08 | 6.26 | 86.88 | 0.65 | 2.01 | 0.32 | 6.07 | 1.33 |
| New | *S. subula* | 42.25 | 14.15 | 77.69 | 0.51 | 3.28 | 0.15 | 6.09 | 1.43 |
| New | *S. subula* | 27.88 | 5.88 | 28.81 | 0.44 | 0.80 | 0.55 | 6.14 | 1.41 |
| New | *S. subula* | 22.61 | 5.27 | 48.92 | 0.43 | 1.11 | 0.39 | 6.63 | 1.44 |
| Old | *S. subula* | 42.13 | 10.34 | 56.82 | 0.26 | 2.39 | 0.11 | 4.14 | 1.02 |
| Old | *S. subula* | 46.71 | 14.23 | 75.39 | 0.76 | 3.52 | 0.22 | 4.88 | 1.22 |
| Old | *S. subula* | 29.31 | 5.62 | 26.23 | 0.52 | 0.77 | 0.68 | 5.30 | 1.27 |
| Old | *S. subula* | 48.52 | 10.58 | 31.96 | 0.70 | 1.55 | 0.45 | 5.31 | 1.36 |
| Old | *S. subula* | 39.51 | 7.86 | 26.22 | NA | 1.04 | NA | 5.38 | 1.38 |
| Old | *S. subula* | 28.77 | 6.86 | 29.75 | 0.58 | 0.86 | 0.67 | 5.51 | 1.33 |
| Old | *S. subula* | 44.66 | 12.49 | 39.42 | 0.55 | 1.76 | 0.31 | 5.52 | 1.31 |
| Old | *S. subula* | 48.36 | 9.42 | 29.61 | NA | 1.43 | NA | 5.55 | 1.30 |
| Old | *S. subula* | 47.17 | 16.72 | 34.64 | 0.47 | 1.63 | 0.29 | 5.58 | 1.28 |
| Old | *S. subula* | 29.26 | 6.07 | 24.09 | 0.49 | 0.70 | 0.69 | 5.62 | 1.32 |
| Old | *S. subula* | 42.74 | 9.73 | 39.69 | 0.48 | 1.70 | 0.28 | 5.65 | 1.30 |
| Old | *S. subula* | 40.14 | 10.91 | 36.92 | 0.47 | 1.48 | 0.31 | 5.68 | 1.34 |
| Old | *S. subula* | 46.34 | 14.1 | 36.42 | 0.47 | 1.69 | 0.28 | 5.71 | 1.32 |
| Old | *S. subula* | 37.43 | 10.7 | 35.72 | 0.50 | 1.34 | 0.38 | 5.72 | 1.37 |
| Old | *S. subula* | 48.72 | 13.23 | 40.09 | 0.63 | 1.95 | 0.32 | 5.80 | 1.34 |
| Old | *S. subula* | 34.23 | 9.43 | 32.15 | NA | 1.10 | NA | 5.81 | 1.42 |
| Old | *S. subula* | 40.68 | 12.93 | 41.95 | 0.63 | 1.71 | 0.37 | 5.87 | 1.33 |
| Old | *S. subula* | 40.53 | 14.88 | 50.02 | 0.64 | 2.03 | 0.32 | 5.87 | 1.41 |
| Old | *S. subula* | 30.78 | 7.4 | 25.14 | 0.42 | 0.77 | 0.54 | 5.90 | 1.38 |
| Old | *S. subula* | 42.75 | 9.99 | 31.25 | 0.48 | 1.34 | 0.36 | 5.91 | 1.39 |
| Old | *S. subula* | 27.93 | 6.67 | 34.03 | 0.58 | 0.95 | 0.61 | 5.94 | 1.39 |
| Old | *S. subula* | 37.72 | 8.42 | 41.89 | 0.62 | 1.58 | 0.39 | 6.15 | 1.47 |
| Old | *S. subula* | 40.37 | 10.7 | 46.28 | 0.70 | 1.87 | 0.37 | 6.38 | 1.42 |
| Old | *S. subula* | 52.64 | 23.85 | 61.56 | 0.66 | 3.24 | 0.20 | 6.49 | 1.49 |
| Old | *S. subula* | 46.18 | 17.17 | 78.1 | 0.72 | 3.61 | 0.20 | 6.50 | 1.50 |
| Old | *S. subula* | 42.63 | 9.41 | 30.72 | 0.44 | 1.31 | 0.34 | 6.61 | 1.49 |
| Old | *S. subula* | 37.58 | 10.88 | 42.34 | 0.27 | 1.59 | 0.17 | 6.64 | 1.56 |
| New | *C. inflexa* | 28.93 | 8.46 | 62.94 | 2.16 | 1.82 | 1.18 | 5.63 | 3.31 |
| New | *C. inflexa* | 30.51 | 7.99 | 64.27 | 2.14 | 1.96 | 1.09 | 5.46 | 3.93 |
| New | *C. inflexa* | 35.46 | 11.54 | 61.97 | 2.55 | 2.20 | 1.16 | 5.92 | 3.53 |
| New | *C. inflexa* | 27.25 | 7.77 | 59.98 | 1.84 | 1.63 | 1.12 | 5.59 | 3.69 |
| New | *C. inflexa* | 22.34 | 4.91 | 62.68 | 1.57 | 1.40 | 1.12 | 5.66 | 3.61 |
| New | *C. inflexa* | 30.6 | 7.96 | 67.99 | 2.54 | 2.08 | 1.22 | 5.80 | 3.73 |
| New | *C. inflexa* | 20.53 | 4.51 | 59.79 | 1.02 | 1.23 | 0.83 | 5.60 | 3.36 |
| New | *C. inflexa* | 19.06 | 4.55 | 61.73 | NA | 1.18 | NA | 5.73 | 3.89 |
| New | *C. inflexa* | 27.36 | 6.43 | 57.79 | 1.84 | 1.58 | 1.16 | 5.68 | 3.30 |
| New | *C. inflexa* | 20.43 | 4.34 | 55.04 | 1.12 | 1.12 | 1.00 | 5.52 | 3.41 |
| New | *C. inflexa* | 19.39 | 4.13 | 58.77 | 1.29 | 1.14 | 1.13 | 5.61 | 3.41 |
| New | *C. inflexa* | 19.35 | 4.86 | 54.27 | NA | 1.05 | NA | 5.27 | 3.89 |
| New | *C. inflexa* | 37.37 | 13.29 | 59.58 | 2.63 | 2.23 | 1.18 | 5.48 | 3.56 |
| New | *C. inflexa* | 21.23 | 6.39 | 67.63 | 1.98 | 1.44 | 1.38 | 5.78 | 3.39 |
| New | *C. inflexa* | 21.96 | 6.38 | 56.88 | 1.30 | 1.25 | 1.04 | 5.26 | 3.75 |
| New | *C. inflexa* | 36.29 | 13.48 | 52.49 | 2.66 | 1.90 | 1.40 | 5.58 | 4.62 |
| New | *C. inflexa* | 26.45 | 7.36 | 53.28 | 1.71 | 1.41 | 1.21 | 5.25 | 4.20 |
| New | *C. inflexa* | 47.69 | 21.61 | 81.13 | 3.93 | 3.87 | 1.02 | 5.85 | 4.73 |
| Old | *C. inflexa* | 22.68 | 8.75 | 64.97 | 1.78 | 1.47 | 1.21 | 5.79 | 4.16 |
| Old | *C. inflexa* | 22.3 | 7.66 | NA | 1.20 | NA | NA | NA | NA |
| Old | *C. inflexa* | 35.02 | 13.62 | 68.09 | 3.11 | 2.38 | 1.31 | 6.03 | 4.41 |
| Old | *C. inflexa* | 24.24 | 10.15 | NA | NA | NA | NA | NA | 4.06 |
| Old | *C. inflexa* | 35.66 | 13.39 | 67.85 | 3.06 | 2.42 | 1.26 | 6.10 | 4.43 |
| Old | *C. inflexa* | 26.86 | 9.26 | 58.77 | 1.93 | 1.58 | 1.22 | 5.38 | 3.99 |
| Old | *C. inflexa* | 29.45 | 11.5 | 55.94 | 1.96 | 1.65 | 1.19 | 5.34 | 4.08 |
| Old | *C. inflexa* | 24.51 | 9.75 | 50.47 | 1.59 | 1.24 | 1.29 | 5.25 | 3.58 |
| Old | *C. inflexa* | 25.8 | 9.03 | 68.15 | 1.80 | 1.76 | 1.02 | 6.08 | 4.31 |
| Old | *C. inflexa* | 27.83 | 11.16 | 54.17 | NA | 1.51 | NA | 5.40 | 3.84 |
| Old | *C. inflexa* | 36.43 | 13.73 | 52.07 | 2.17 | 1.90 | 1.14 | 5.23 | 4.04 |
| Old | *C. inflexa* | 40.16 | 14.65 | 65.23 | 3.37 | 2.62 | 1.28 | 6.14 | 4.59 |
| Old | *C. inflexa* | 27.28 | 9.92 | 56.82 | 1.85 | 1.55 | 1.19 | 5.57 | 3.95 |
| Old | *C. inflexa* | 34.18 | 12.32 | 58.76 | 2.52 | 2.01 | 1.25 | 5.58 | 4.40 |
| Old | *C. inflexa* | 35.61 | 14.16 | 71.35 | 3.07 | 2.54 | 1.21 | 6.04 | 4.38 |
| Old | *C. inflexa* | 22.78 | 6.58 | 64.61 | 1.65 | 1.47 | 1.12 | 5.40 | 4.29 |
| Old | *C. inflexa* | 27.42 | 9.48 | 62.34 | 1.97 | 1.71 | 1.15 | 5.65 | 4.13 |
| Old | *C. inflexa* | 34.46 | 15.59 | 73.44 | 3.14 | 2.53 | 1.24 | 6.22 | 4.79 |
| Old | *C. inflexa* | 33.44 | 15.26 | 57.13 | 2.36 | 1.91 | 1.23 | 5.19 | 4.02 |
| Old | *C. inflexa* | 21.3 | 6.72 | 60.79 | 1.63 | 1.29 | 1.26 | 5.59 | 4.11 |
| Old | *C. inflexa* | 36.34 | 15.72 | 59.89 | 2.74 | 2.18 | 1.26 | 5.53 | 4.16 |
| Old | *C. inflexa* | 20.51 | 9.96 | 49.39 | 1.46 | 1.01 | 1.44 | 4.91 | 3.30 |
| Old | *C. inflexa* | 30.48 | 12.29 | 63.63 | 2.44 | 1.94 | 1.26 | 5.78 | 3.93 |
| Old | *C. inflexa* | 25.24 | 7.71 | 62.69 | 2.35 | 1.58 | 1.49 | 5.48 | 3.88 |
| Old | *C. inflexa* | 27.82 | 8.66 | 63.48 | 3.68 | 1.77 | 2.09 | 5.45 | 4.25 |
| Old | *C. inflexa* | 24.26 | 7.76 | 59.19 | 1.79 | 1.44 | 1.25 | 5.81 | 3.98 |
| Old | *C. inflexa* | 28.61 | 8.62 | 57.22 | 2.04 | 1.64 | 1.25 | 5.53 | 4.24 |
| Old | *C. inflexa* | 33.24 | 13.34 | 60.69 | 2.76 | 2.02 | 1.37 | 5.97 | 3.80 |
| Old | *C. inflexa* | 26.58 | 12.4 | 55.87 | 2.22 | 1.49 | 1.50 | 5.31 | 4.10 |
| Old | *C. inflexa* | 30.83 | 14.89 | 51.98 | 3.10 | 1.60 | 1.93 | 5.09 | NA |
